# Evolution of the Development of Core Competencies in Pharmaceutical Medicine and Their Potential Use in Education and Training

**DOI:** 10.3389/fphar.2020.00282

**Published:** 2020-03-19

**Authors:** Peter D. Stonier, Honorio Silva, Alan Boyd, Domenico Criscuolo, Felicity J. Gabbay, Kyoko Imamura, Gustavo Kesselring, Sandor Kerpel-Fronius, Heinrich Klech, Ingrid Klingmann

**Affiliations:** ^1^Faculty of Pharmaceutical Medicine, Royal College of Physicians, London, United Kingdom; ^2^International Federation of Associations of Pharmaceutical Physicians and Pharmaceutical Medicine, Woerden, Netherlands; ^3^PharmaTrain Federation, Brussels, Belgium

**Keywords:** pharmaceutical medicine, syllabus, curriculum, core competencies, PharmaTrain, IFAPP, Faculty of pharmaceutical medicine

## Abstract

The evolution of postgraduate vocational education and training in pharmaceutical medicine is described alongside the growth of this scientific-medical discipline and profession for the development of new medicines. Over the past 50 years, whilst the training of competent professionals for their work has been paramount, this has paralleled the need to engage with the rapid and complex changes in R&D technologies, patient and healthcare system needs, and the ethical and regulatory obligations applied to the development of medicines throughout their lifecycle. The move from unstructured training to formal programs with syllabus, curricula and assessments for certification, has been accompanied by educational changes to outcomes-based, learner-centered, competency-based programs. The evolution of education and training along with the development of the set of 57 core competencies for professional practitioners in pharmaceutical medicine are described within the competence framework of seven domains: discovery of medicines and early development; clinical development and clinical trials; medicines regulation; drug safety and surveillance; ethics and subject protection; healthcare marketplace; communication and management. The application of the core competencies in a harmonized, international platform of education and training in medicines development at the undergraduate, postgraduate and continuing professional development levels would invigorate the potential for having a competent workforce with the intent to provide faster access to better and appropriate medicines for patients worldwide.

## Background

Pharmaceutical medicine is the medical scientific discipline concerned with the discovery, development, evaluation, registration, monitoring and medical aspects of marketing of medicines for the benefit of patients and the public health (Faculty of Pharmaceutical Medicine, 2019).

The employment of doctors in medical departments and R&D operations of pharmaceutical companies, the need for clinicians and prescribers to be involved in the development of marketed medicines, the increased influence of payers and providers, the proven needs of involving patients in their treatment, and the need to consider clinical and cost effectiveness in making medicines available to patients and the public, have all served to increase the engagement of the biomedical profession in the development, introduction and maintenance of medicines (Cromie, 1993; Gabbay, 2003; Young and Stonier, 2011). Over the last 50 years pharmaceutical medicine evolved from these needs for medical engagement in the development and commercialization of medicines to become a broad scientific-medical discipline. In some countries and territories pharmaceutical medicine remains as an adjunct discipline, in some others it has become a formal, legal medical specialty as part of the medical profession. Pharmaceutical medicine is thus a global and multi-disciplinary field, involving in its work a wide range of biomedical graduates, scientific and technical groups together along with support from administrative, legal and financial professions. The term ‘medicines development science’ is used alongside ‘pharmaceutical medicine’ to acknowledge this multi- professional discipline.

The academic system has been historically slow in recognizing the needs for formal postgraduate education in medicines development and thus the first initiatives were left to the professional bodies.

A group of pioneer pharmaceutical physicians in the United Kingdom came together to form the Association of Medical Advisers in the Pharmaceutical Industry in 1957 (AMAPI, later British Association of Pharmaceutical Physicians, BrAPP) as a vehicle for mutual support and, for a long period, to promote non-structured education and training for its members.

Pharmaceutical medicine was organized as a novel discipline through the creation of the International Federation of Associations of Pharmaceutical Physicians (IFAPP) in 1975 followed by the introduction of the first structured training program organized by University of Wales Institute for Science and Technology (UWIST; now Cardiff University), under guidance from a joint committee of AMAPI and the Association of the British Pharmaceutical Industry (ABPI). This program evolved and in 1978 became the *Postgraduate Course in Pharmaceutical Medicine* (Luscombe and Salek, 2001).

These initiatives paralleled the creation of the *Diploma in Pharmaceutical Medicine (DPM)* an examination- based certification established in 1976 by the Royal Colleges of Physicians of the United Kingdom aiming to advance the discipline and to establish standards (Binns, 1976; Smith, 2000). These developments were the start of the organized profession of pharmaceutical medicine.

This was followed by many countries with professional associations encouraging the creation of vocational postgraduate education and training. The incorporation of 30 national associations was coordinated through IFAPP, mostly concerned with the recognition of pharmaceutical medicine as a medical specialty at the national level and fostering initiatives for postgraduate education and continuing professional development (CPD). As a result, a limited number of postgraduate programs are offered in a few countries in Western and Central-Eastern Europe, Latin America and Asia with relative success.

The early training programs and courses in pharmaceutical medicine followed a Syllabus of topics across the discipline. The Syllabus in Pharmaceutical Medicine was derived from an understanding of the knowledge concerning the clinical testing and licensing of new medicines and their introduction into medical practice and was developed for the *Postgraduate Course in Pharmaceutical Medicine*. The knowledge-based Syllabus, essentially a list of topics defining the universe of knowledge in and boundaries of pharmaceutical medicine, was developed initially in the United Kingdom and had frequent revisions as a result of the evolving discipline, to incorporate newer topics and to re-emphasize others (Shelley, 1991; Stonier and Gabbay, 1992). With the PharmaTrain project (see below) the opportunity was taken to develop the Syllabus as a global, aligned syllabus for these knowledge-based initiatives. The current version is the PharmaTrain Syllabus in Pharmaceutical Medicine/Medicines Development Science (V2.0 January 2018) and is recommended as a platform for global education and training (Faculty of Pharmaceutical Medicine, 2019).

## Toward the Development of Certification Through a Competency-Based Training Curriculum

Recognizing the growing acceptance of the discipline among medical professionals, BrAPP proposed that the UK Royal College of Physicians establish a Faculty of Pharmaceutical Medicine. The Faculty was inaugurated in 1989 as a new professional group and standard-setting body representing the medical discipline of pharmaceutical medicine in the United Kingdom (Goldberg and Smith, 1985). The foundation of the Faculty established the need for entry criteria for new members and reconciled training needs for entry via its certification program, the *Diploma in Pharmaceutical Medicine-DPM*, granted by the Royal Colleges of Physicians of the United Kingdom.

There was however a concern that the academic knowledge base itself was not a total reflection of the professional attributes required to practice safely within the scope of a newly proposed medical specialty, and that specific personal skills and business management knowledge should be recognized and incorporated into training programs (Stevens, 1987).

In 1992 a survey was conducted amongst the 810 combined membership of the Faculty and BrAPP. The survey aimed to identify items of knowledge and skills considered important in the daily work of pharmaceutical physicians, to identify training needs and timing, and to explore the relationship between training needs and actual training received. An outcome of this survey led to opportunities to develop new training programs, as a few items of knowledge and skills were identified which were considered important to practitioners but for which there was a shortfall in training. Such items fell across the specialty medical and technical areas of medicines’ development, but the majority were related to personal transferable skills and to business management (Stonier and Gabbay, 1992). The outcomes of this survey also served as a foundation to define the professional competencies.

The Faculty and Joint Committee on Higher Medical Training (JCHMT) of the Royal Colleges of Physicians worked together to pursue the medical specialty recognition for pharmaceutical medicine and the introduction of a specialist training program. As a result, Pharmaceutical Medicine Higher Medical Training (HMT) was designed around six practical domains with continuous assessment. HMT would be an accredited vocational program in order to meet local opportunities for direct in-workplace experience and training, and for indirect training through interactive external courses.

The proposed HMT program comprised two parts, basic HMT and advanced HMT, with the individualized program completed in an indicative 4-year period. Basic HMT included the knowledge and applied knowledge (cognitive competency) described in the Syllabus for Pharmaceutical Medicine. This was assessed through the examination for the DPM, which remained the assessment of the knowledge- based component for HMT and for the subsequently titled Pharmaceutical Medicine Specialty Training program (PMST).

Advanced HMT was a workplace-based experiential program covering six domains of practice within pharmaceutical medicine: regulation of medicines, early and late clinical development, data management and statistics, drug safety and surveillance. A further domain of interpersonal, management and leadership matters was recognized and added to address general transferable skills applicable to practice in pharmaceutical medicine.

The Faculty produced draft outlines for the six practice-based specialty modules and in 1998 commissioned the University of Keele to undertake a Delphi exercise to determine their content (Millson et al., 2000).

There was a total of 364 statements of knowledge and skills at the start of the Delphi process, and these were gathered to form the six curricular domains with a level of achievement given for each constituent competency. The outcomes of each Delphi exercise on the six modules were analyzed at the University of Keele and published (Commentary, 1999; Millson et al., 1999; Phelan et al., 1999; Boardman et al., 2000).

With the results of the Delphi exercise for the competencies in each of the six specialty domains the Faculty’s Specialist Training Committee developed and added knowledge, skills and attitudes/behaviors statements to the competencies to form the curricular content of six practical modules in advanced training in pharmaceutical medicine (Faculty of Pharmaceutical Medicine, 1999; Faculty of Pharmaceutical Medicine, 2000).

This competency-based curriculum was a relatively recent innovation and had benefited from new outcomes-based educational techniques. Commentators at the time noted that few medical specialties had been so original in determining the curriculum for the training of their specialists (Editorial, 2000).

In parallel with the development of the competency-based workplace-centered HMT program, steps were taken through the Faculty and JCHMT to obtain formal, legal listing of pharmaceutical medicine as a medical specialty in Schedule 2 of The European Specialist Medical Qualifications Order 1995, restricted to the United Kingdom. This was achieved on April 17, 2002.

The advances in education and training in the United Kingdom were closely followed by IFAPP. The IFAPP Council for Education in Pharmaceutical Medicine (CEPM) was established in 2002 to promote education in pharmaceutical medicine in collaboration with its member associations, to advise new and established members on setting up courses and examinations, to harmonize established courses for diplomas in pharmaceutical medicine, and to foster the recognition of pharmaceutical medicine as a medical scientific discipline.

## Further Development of the Curriculum in United Kingdom; Pharmaceutical Medicine Specialty Training (PMST)

In 2005, the medical regulator’s intent was to replace HMT with a new updated curriculum developed with all medical specialties. The new curriculum was renamed *Pharmaceutical Medicine Specialty Training* (PMST) as a vocational program aimed to achieve competence in the workplace.

The PMST curriculum covering knowledge, assessed through the Diploma in Pharmaceutical Medicine examination, practical competencies and generic aspects was designed for training of pharmaceutical physicians who enter the specialty of pharmaceutical medicine after four years of post-qualification clinical training and experience.

PMST is available for doctors working in pharmaceutical companies, clinical research organizations, academic clinical research units or regulatory bodies to gain a *Certificate of Completion of Training* (CCT) from the medical regulator, the General Medical Council.

In addition to some updated competencies, reflecting experience in practice, and clarification of terminology of knowledge, skills and attitudes/behaviors the new PMST included workplace-based assessments, and an e-portfolio to record evidence, assessments, achievement and progress in the training program. PMST is thus a competency-based program delivered through the workplace, known as the Local Educational Provider (LEP), together with an Educational Supervisor (mentor) allocated to each trainee.

In 2019 there are 360 specialist pharmaceutical physicians who have completed PMST and have a place on the GMC’s specialist register in pharmaceutical medicine. There are 140 physicians enrolled currently in PMST working in over 60 LEPs approved for training.

Whilst the PMST program might satisfy the present requirements of the curriculum for specialist training in the United Kingdom, vocational education and training is a dynamic process, subject to change as a result of evolving working practices and new skills with professional requirements which might transform both the curricular domains and their constituent competencies.

## Development of Core Competencies in Pharmaceutical Medicine. the Role of IFAPP and Pharmatrain

The development of a competency-based curriculum in the discipline of pharmaceutical medicine depended firstly on agreeing the broad domains relevant to the field and within them defining and building the competencies with the knowledge, skills and attitudes/behaviors which meet the objective of the competency and are also mapped to the knowledge-based Syllabus for Pharmaceutical Medicine.

With the advent of competency-based education there was a realization within IFAPP of the need to develop and maintain a list of core competencies to meet the requirements of the profession, and a responsibility to orientate and focus the discipline and related academic programs for the development of competent professionals and influence the profession of pharmaceutical medicine (Stonier et al., 2007).

Twenty eight IFAPP member associations when surveyed showed that only 20% of their membership had received postgraduate education in pharmaceutical medicine (Silva et al., 2012, 2013). Similarly, surveys conducted in the United States among pharmaceutical physicians revealed that the respondents lacked formal training in critical areas of drug development (Stonier et al., 2011).

One response to this lack of education and training was a call from the largest public-private partnership in biomedicine, the Innovative Medicines Initiative (IMI), to integrate existing expertise and further raise the quality of postgraduate education and training in pharmaceutical medicine for all professionals working in medicines development. In 2009 the ‘PharmaTrain’ project was awarded to a consortium of all European academic providers of pharmaceutical medicine courses, IFAPP, and experts from not-for-profit organizations and other universities offering training in this discipline. They collaborated with experts from the participating pharmaceutical companies on development of the PharmaTrain Syllabus (V1.0. 2010), in turn adapted from the Faculty, as well as harmonized curricula for a modular diploma base course, a master’s degree and a CPD platform, aiming to align the opportunities for education and training in Europe. Nine quality criteria for course providers were defined and a course recognition system developed and implemented. The harmonized quality training program for post-graduate education in Medicines Development is now applied by PharmaTrain-recognized academic and training organizations in Europe and worldwide (Klech et al., 2012; PharmaTrain Manual, 2012).

Based on these activities, there is now firmer collaboration between all parties involved. A working group to start accruing core competencies was established within IFAPP’s CEPM including representatives from PharmaTrain, academic institutions and IFAPP’s member associations, with special interest and experience on quality improvement through education. A review and analysis of the core competencies published by academic groups or professional associations relating to pharmaceutical medicine was undertaken. A combination of bibliographic search and consultation with related groups was agreed, using a modified six- sigma approach to process improvement. The domains were identified through benchmarking, alignment and harmonization of domains and competencies from similar or related groups. The competencies developed by the Faculty formed the foundation for this exercise (Faculty of Pharmaceutical Medicine, 2010).

The critical issues considered were the areas and domains for competence, and their intrinsic and extrinsic validity; the descriptors for each competency and their relevance; the level of granularity and comparability with other disciplines and professions, and the level of anticipated expertise. The group focused on the cognitive aspects for each proposed competency and conducted a mapping exercise with the learning outcomes and curriculum for the PharmaTrain Diploma base course. The competencies were verbalized using the highest wording associated with the competence category in the revised Bloom’s Taxonomy (Bloom, 1956). The final version of the core competencies was authorized at IFAPP’s General Assembly in Barcelona, Spain November 17, 2012.

Resulting from this, seven domains of competencies were identified within the competence framework: discovery of medicines and early development; clinical development and clinical trials; medicines regulation; drug safety and surveillance; ethics and subject protection; healthcare marketplace; communication and management.

A total of 57 core competencies for pharmaceutical physicians and drug development scientists was included. The learning outcomes of the PharmaTrain Diploma base course were aligned (93%) with the competencies.

**FIGURE 1:**
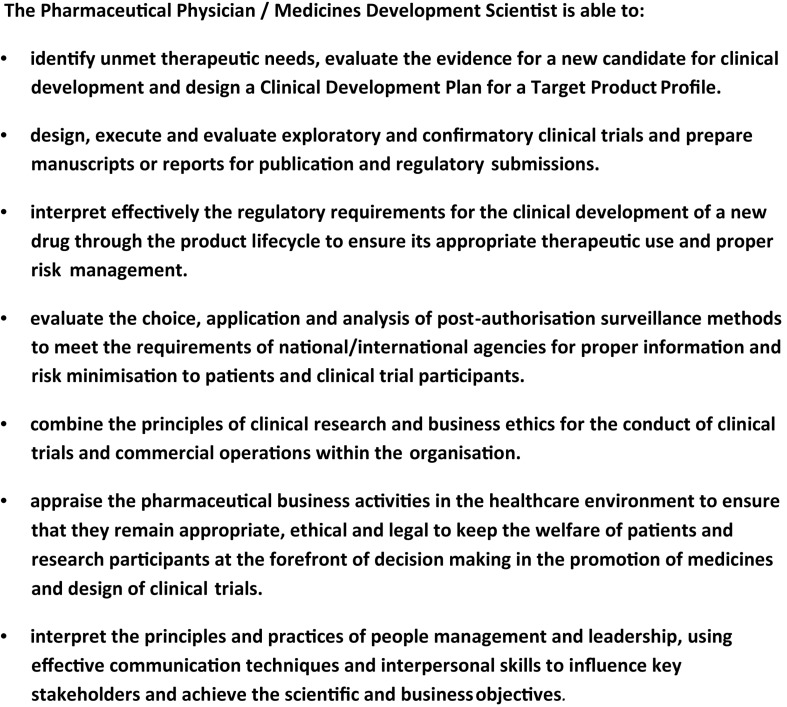
Statement of competence in pharmaceutical medicine/medicines development science.

A Statement of Competence summarizing the competency domains was prepared (Figure 1). This is a concise description for a competent professional who can contribute to any stage of product life-cycle management (Silva et al., 2013).

From January 2015 to March 2016 the renamed IFAPP-PharmaTrain competency working group (IPCWG) revised the core competencies in a process like that conducted previously. The skills and behaviors associated with the applied knowledge for each of the core competencies were identified. IFAPP member associations engaged in a consultation exercise with the draft version, before a final set of full core competencies was agreed and adopted at the IFAPP House of Delegates Meeting in São Paulo in April 2016 (see Supplementary Annex 1).

The core competencies were revised and updated by the IPCWG in 2018, and as a result a few changes in skills and behaviors were included.

## Intended Use of the Core Competencies in Pharmaceutical Medicine

The core competencies can serve as a resource and guide for improving the accountability and quality of education and training in pharmaceutical medicine.

They were developed recognizing the distinctiveness and diversity in the complex world of medicines’ development. The model may foster further detailed development and identification of sub-competencies that might be applicable to specific functions in clinical research and drug development.

The primary vision for this competency model is the availability of professionals who are more fully prepared to meet the challenges and opportunities in pharmaceutical medicine/medicines development science in the next decade.

Competency-based profiles of key jobs in medicines development can be prepared, and standardized job descriptions for different functions could be developed globally.

The effective implementation of training programs using the core competencies anywhere in the world may renovate drug development to be an efficient process integrated with product lifecycle management and resulting in the availability of better medicines. Several competency-based programs are currently in the planning phase (Chisholm, 2019; Schnetzler et al., 2019).

A knowledge-based online program *‘Medical Affairs in Medicines Development’* sponsored by IFAPP and King’s College London is now available to students worldwide. Its learning outcomes are aligned with the core competencies^1^.

Developments of competency models are iterative processes, and the model described here will have to be updated regularly as the competencies are employed for professional, academic or self-assessment purposes. Continued dialogue regarding the use of the competencies, their relevance, and ongoing changes in the fields of pharmaceutical medicine and related drug development sciences will make the changes imperative. Competency sets generally have a lifespan of 3–5 years (Batalden et al., 2002; Calhoun et al., 2008).

Professional groups elsewhere in clinical research are working to define the roles and competencies of individuals working in specific areas, including physician investigators, nurses, investigational site staff as well as other professions involved in regulatory affairs, project management, translational science and comparative effectiveness (Koren et al., 2011; Jones et al., 2012; Sonstein et al., 2014; The Global Health Network, 2016; Calvin-Naylor et al., 2017; ACRP, 2019).

The set of core competencies, together with the PharmaTrain Syllabus, serve as a guide to the IFAPP member associations and related institutions worldwide to develop undergraduate, postgraduate and CPD programs in pharmaceutical medicine/medicines development science.

## Author Contributions

All authors contributed both to the development of the ideas as well as to the writing of the manuscript and the linked Supplementary Material.

## Conflict of Interest

The authors declare that the research was conducted in the absence of any commercial or financial relationships that could be construed as a potential conflict of interest.

## Footnotes

1 www.ifappacademy.org
