# Supplementary material for: Evolution of the Development of Core Competencies in Pharmaceutical Medicine and Their Potential Use in Education and Training
Source: Front Pharmacol. 2020 Mar 19;11:282. doi: 10.3389/fphar.2020.00282 (PMC7096542; doi:10.3389/fphar.2020.00282)

| **IFAPP-PHARMATRAIN FEDERATION COLLABORATION WORKING GROUP*** |
| --- |
| **CORE COMPETENCIES IN PHARMACEUTICAL MEDICINE AND MEDICINES DEVELOPMENT** |
| Applied Knowledge, Skills and Behaviours |
| **Version 1.2** |
| **OCTOBER 18, 2018** |

***Working Group Members:**

| ***Domenico Criscuolo*** | ***Dominique Dubois*** | ***Stewart Geary*** | ***Kyoko Imamura*** |
| --- | --- | --- | --- |
| ***Sandor Kerpel-Fronius*** | ***Gustavo Kesselring*** | ***Heinrich Klech*** | ***Ingrid Klingmann;*** |
| ***Greg Koski*** | ***Xavier Luria*** | ***Joao Massud*** | ***Gerfried Nell*** |
| ***Rudolf van Olden*** | ***Beate Schmidt*** | ***Honorio Silva (Chair)*** | ***Peter Stonier (Co-Chair)*** |

**Area 1:**

**Drug Development & Clinical Trials**

# Domain 1: Discovery of Medicines and Early Development

## The Pharmaceutical Physician / Medicines Development Scientist is able to identify unmet therapeutic needs, evaluate the evidence for a new candidate for clinical development and design a Clinical Development Plan for a Target Product Profile.

**C1: EVALUATION AND ANALYSIS OF A DISEASE AREA WITHIN THE INDUSTRY CLINICAL DEVELOPMENT ENVIRONMENT AND IDENTIFICATION OF UNMET THERAPEUTIC NEEDS.**

**Applied Knowledge of:**

- The causative factors, pathophysiology and main therapeutic options in one major organ-based disease.
- The benefits and shortcomings of current therapy, thereby identifying new therapeutic needs in one major organ-based disease.
- How advancing knowledge of molecular biology, pharmacogenomics and pharmacogenetics, may tailor therapy.
- How to evaluate and interpret the literature findings in the clinical development environment.

## Skills:

- To bring together scientists working on the underlying disease process, including academic and company experts on treatment options, and other specialists developing new compounds that may fulfil unmet needs.
- To contribute to proposed investigations and profiling of a new theoretical agent by applying key principles of efficacy, safety and economic value.
- To conduct a clinical literature search.
- To write or review a brief report describing:
  - the epidemiology and pathophysiology and natural history of disease area;
  - efficacy and safety evidence of therapies available and their mechanisms of action;
  - a summary of products under development in this area;
  - unmet medical / therapeutic need in this area;
  - standards of care in this area.

## Behaviours:

- As part of a research team, consults with academic and clinical experts in the therapeutic area to learn therapeutic unmet needs, aims, and achievements.
- Recognises constraints in clinical practice and in healthcare provisions.
- Recognises the breadth and depth of data requirements and the inherent limitations of information freely available in the public domain when making appropriate clinical development judgements.
- Works as part of a team to ensure the fullest understanding of non-clinical, clinical and commercial data, and their relevance to the disease area review.

**C2. EVALUATION OF THE CLINICAL AND NON-CLINICAL PHARMACOLOGY AND TOXICOLOGY**

## EVIDENCE FOR A NEW CANDIDATE FOR CLINICAL DEVELOPMENT.

**Applied Knowledge of:**

- Understanding the non-clinical tests of a candidate drug’s pharmacology and toxicology and their limitations.
- ICH Topic M3, Non-Clinical Safety Studies for the Conduct of Human Clinical Trials and Marketing Authorisation for Pharmaceuticals.
- The clinical significance of in vitro and in vivo animal pharmacology.
- Standard animal toxicology study designs and toxicokinetics.
- Animal: Human toxicity concordance, species variability.
- Differences in the non-clinical evaluation of small chemical molecules and biological medicinal products.
- The research, studies and data that should be available to make an informed decision to proceed to clinical efficacy studies (Phase II).
- The clinical significance of biomarkers in translating animal pharmacological effects to humans.
- The clinical significance of biopharmaceutical formulation for optimal drug administration.

## Skills:

- To understand the evidence for a candidate investigational product’s potential value from non-clinical studies in various species, either whole animal or isolated organ and tissue models, and in models of disease.
- To relate longer-term animal toxicology to the potential therapeutic indications and dosages.
- To use preclinical metabolism data to identify necessary clinical drug interaction studies.
- To interpret and evaluate the safety of an investigational product in order to mitigate risk, and plan a safe clinical development programme.
- To review, evaluate and discuss the safety and toxicology data for a new drug candidate (real or hypothetical) planned for a clinical trial programme, from the First-in-Human (FIH) studies onwards.
- To understand the functional significance of biomarkers in the translational process from animals to human.
- To appreciate the effect of biopharmaceutical formulation on the effective delivery of drugs to the right place.

## Behaviours:

- As a therapeutic / development team member, contributes to the stepwise decisions being made based on non-clinical pharmacology and toxicology from the perspective of therapeutic needs and patient safety.
- Recognises the benefits and pitfalls of extrapolating preclinical data to the predictions of drug effects in man.
- Communicates the relevance of the non-clinical data to others working on the investigational product’s development.
- Explain to clinicians the functional significance of biomarkers in clinical trials.
- Explain to clinicians the functional significance of biopharmaceutical formulation for the therapeutic application of medicines.

**C3. EVALUATION AND APPLICATION OF THE REGULATORY AND ETHICAL ASPECTS**

## UNDERPINNING CLINICAL DEVELOPMENT.

**Applied Knowledge of:**

- The principles of the Declaration of Helsinki and the Belmont Report.
- The ethical issues that might arise from clinical trials.
- Regulations governing clinical trials in the various regions and countries e.g. WHO, EU-Clinical Trials Directive Regulation.
- The requirements of ICH Good Clinical Practice.
- International databases, registries and repositories.

## Skills:

- To predict and address the ethical issues arising from clinical studies.
- To draft or review an informed consent form (ICF) that includes all the ICH-required elements, written in appropriate, patient-friendly language.
- To write or review the clinical section of a Clinical Trial Application (CTA) and / or an IND.
- To describe how the country/regional regulatory authority view and apply the Declaration of Helsinki.

## Behaviours:

- Recognises the ethical environment in which clinical trials are conducted and the contribution made by patients in agreeing to participate in clinical research.
- Recognises that clinical development is a global process regulated by differing regional frameworks.
- Ensures as part of the development team that ethical issues arising in clinical development are fully understood.

**C4. CREATION OF A CLINICAL DEVELOPMENT PLAN (CDP) FOR A NEW CANDIDATE INCLUDING**

## A TARGET PRODUCT PROFILE (TPP).

**Applied Knowledge of:**

- The elements of a Clinical Development Plan, including:
  - The key studies required for registration.
  - The primary and secondary endpoints.
  - Timelines for study and programme completion.
  - Possible risks that would threaten the plan.
- The objectives of studies in support of the product’s expected lifecycle.

## Skills:

- To write or contribute to a Clinical Development Plan (CDP) for a new drug (real or hypothetical).
- To create an idealised Target Product Profile (TPP).

## Behaviours:

- Recognises the role, value and benefit of the team approach to clinical development and recognises the contribution of others to the successful CDP.
- Recognises the importance of an integrative view on the entire clinical development process.

**C5. THE DESIGN AND EXECUTION OF EXPLORATORY STUDIES AND EVALUATION OF THE**

## RESULTING DATA AS APPLIED TO THE CLINICAL DEVELOPMENT PLAN (CDP) AND ACHIEVE A TARGET PRODUCT PROFILE (TPP).

**Applied Knowledge of:**

- The objectives of early phase drug studies in man.
- The rationale, advantages and disadvantages of the use of healthy or patient volunteers in early-phase drug studies.
- Requirements for the inclusion of special populations e.g. elderly, female in early phase studies.
- The objectives and limits to be applied in order to maximise the information obtained and to avoid or minimise risks to study subjects.
- Human pharmacokinetics, pharmacodynamics and pharmacogenetics.
- Selecting dose range and increments relating to minimum effective and maximum tolerated doses.
- Regulatory and legal requirements in human studies.
- The degree of biological variation seen in a normal population.
- The reasons and need for full screening of healthy volunteers.
- The required critical data in a study.
- The special rules in oncology and immunotherapy to design early phase clinical trials.

## Skills:

- To contribute to or review the design of human studies in order to fulfil their aims.
- To review, evaluate and discuss the safety and toxicology data for a new drug candidate (real or hypothetical) planned for a clinical trial programme, from the first-in-human (FIH) studies onwards.
- To recommend, with reasons, on the basis of non-clinical and Phase I data, a range of doses to be studied in Phase II.
- To define the subsequent aims and safeguards in healthy volunteer studies and early patient trials; instil these and monitor compliance.
- To select safety measures based on non-clinical data and related drugs.
- To check and interpret any physiological changes observed.
- To propose or review any dosing changes or limits for subsequent Phase II or Phase III studies.
- To contribute to the design and preparation of a study protocol for a new drug (real or hypothetical).
- To review a number of outline protocols considering:
  - How they achieve the aims of the CDP.
  - How they comply with ethical requirements.
- To recommend and discuss the required characteristics of patients within the CDP.

## Behaviours:

- Recognises the value of healthy volunteer studies in product development and participates actively in their evaluation.
- Recognises responsibilities to study volunteers in order to ensure their safety.
- Imparts this ethos to others and monitors that safeguards are being applied.
- Recommends any actions needed as studies progress, such as stopping planned dose escalation or introducing new safety checks.
- Recognises the importance of a medical input to the evaluation of early development data, and shares this with the product development.

**C6. THE EVALUATION OF THE ADVANCES MADE IN THE CLINICAL PHARMACOLOGY OF A NEW**

**MEDICINE IN A STEPWISE MANNER WITH THE OVERALL CDP AND THE TPP.**

## Applied Knowledge of:

- How, in particular, the pharmacology and toxicology data necessary for Phase 0 and 1 studies must be designed, reviewed and approved in readiness for clinical trials.
- Components of the CDP and other requirements for a successful regulatory or licensing submission worldwide.
- The clinical pharmacology requirements in a regulatory submission for approval of a new medicine and in a Summary of Product Characteristics (SmPC) or its equivalent.
- The application of clinical pharmacological knowledge and methodology in the development programme from choice of a candidate entity through its full characterisation and key decisions on its therapeutic potential and any limitations on its clinical use.
- Causes of variability in human drug response.

## Skills:

- To define or review the planned clinical pharmacology of the candidate investigational product before clinical trials are begun.
- To anticipate possible disease-related variations in drug handling in patients compared with healthy volunteers.
- To react to unexpected findings promptly and, if necessary, suspend further work while other expert opinions are obtained and the issue is clarified.
- To understand past problems in this clinical or therapeutic area that have led to regulatory refusal of trials or their modification.
- To write or review Expert Reports, Clinical Overviews and Product Information.
- To establish in a logical and stepwise manner the main pharmacological actions of a new medicine in healthy people and in those with the target disease.
- To identify the likely dose range and, early on in the programme, measure its clinical effects (proof of concept).
- To identify further studies with other drugs in that therapeutic class aimed to determine their comparative efficacy and pharmacokinetic profile.
- Able to obtain advice on any specific regulatory needs worldwide.
- To anticipate possible adverse interactions with other drugs, which are likely to be co-prescribed for other medical conditions in routine clinical practice, and of impaired pharmacokinetics due to co-existing medical conditions.
- To recommend, with reasons, on the basis of non-clinical and Phase I data, a range of doses to be studied in Phase II.

## Behaviours:

- Accepts a pivotal role in preparation of a development plan that requires knowledge and judgement.
- Recognises the value of liaison with experts in related fields in the design and interpretation of studies.
- Exhibits strict compliance with regulations and guidelines.
- Understands the need to keep senior management informed.
- Recognises the need to characterise pharmacokinetic (absorption, distribution, metabolism and excretion, ADME) studies how a new medicine is handled in the human body.
- Realises that impairment of normal ADME may be caused by the disease itself and/or other drugs likely to be given to treat the disease.
- Communicates the importance of clinical pharmacology to other members of the development team.

**C7. EXPLANATION OF THE STATISTICAL PRINCIPLES FOR THE DESIGN, CONDUCT AND**

**ASSESSMENT OF EXPLORATORY STUDIES.**

**Applied Knowledge of:**

- The fundamentals of applied parametric and non-parametric biostatistics as applied to clinical pharmacology and medicines development.
- The use of control, blinding, randomisation and other methods for the reduction of bias in clinical trials.
- The principles of power and sample size, the reduction of variation and other methods for increasing precision in clinical studies.
- Methods for the interim analysis of clinical trial data and the management of those analyses, through an Independent Data Monitoring Committee, for the evaluation of efficacy, harm and futility.

## Skills:

- To select the most appropriate design structure: superiority; equivalence; non-inferiority; dose-response - in order to meet the needs of the drug development programme.
- To provide clinical input into sample size calculations, the selection of primary and secondary endpoints, and choice of comparator and methods of interim analysis.

## Behaviours:

- Recognises the importance of working with a statistician in the design of clinical studies.

**C8. JUSTIFICATION FOR THE VARIOUS END-POINTS USED IN THE CLINICAL DEVELOPMENT**

**PROGRAMME.**

**Applied Knowledge of:**

- The main imaging techniques used currently in clinical trials.
- The main laboratory methods used in clinical trials.
- The use and examples of biomarkers and surrogate endpoints.
- The use Patient Reported Outcome (PRO) endpoints such as:

-Health Related Quality of Life (HRQL), symptoms, functional status, treatment preferences.

-Relevant guidelines.

## Skills:

- To recommend, with reasons, appropriate imaging, laboratory methods and biomarkers and surrogate endpoints for a study protocol (real or hypothetical).
- To plan the incorporation of PRO assessment in interventional protocols.
- To recommend, with reasons, appropriate patient-reported questionnaires for a study protocol.
- To compare the different types of instruments available to measure health related quality of life.
- To critically review the methodological quality of published PRO studies.

## Behaviours:

- Recognises the contribution of new technologies and approaches in the field of clinical research on new medicines.
- Recognises the time-limitation of publications in a rapidly developing technical field.
- Recognises the contribution of the development and application of PRO instruments in the drug development process, particularly to support product labelling claims.

**C9. APPRAISAL OF SUSPECTED ADVERSE EVENTS DURING EXPLORATORY DEVELOPMENT.**

## Applied Knowledge of:

- The ICH guidelines definition and classification of adverse events and use of MedDRA terminology.
- The working requirements for expedited and aggregate reporting of adverse events at each stage of product development in EU, USA and the rest of the world.
- Principles required for developing REMS (risk evaluation and mitigation strategy) and RMP (risk management plans).

## Skills:

- To evaluate adverse events for seriousness and causality.
- To categorise and ‘report’ some hypothetical examples of adverse events based on patient case histories.
- To be vigilant for identifying adverse events that are not necessarily drug-related, but have been associated historically with adverse reactions for other drugs and are therefore worthy of heightened pharmacovigilance e.g. hepatotoxicity, Stevens-Johnson Syndrome.

## Behaviours:

- Recognises the importance of a thorough evaluation of all emerging safety data as part of the developing safety profile of a product, in order to identify adverse safety signals early and avoid exposing patients to unnecessary risk.

# Domain 2: Clinical Development and Clinical Trials

## The Pharmaceutical Physician / Medicines Development Scientist is able to design, execute and evaluate exploratory and confirmatory clinical trials and prepare manuscripts or reports for publication and regulatory submissions.

**C10. EVALUATION OF THE CONDUCT AND MANAGEMENT OF CLINICAL TRIALS WITHIN THE CONTEXT OF THE CLINICAL DEVELOPMENT PLAN (CDP) AND WORKING AS PART OF A TEAM.**

**Applied Knowledge of:**

- The information and data that would support or preclude the ongoing development of a product.
- The types of adverse effects that are likely to be encountered.
- The clinical pharmacokinetics data that would be gathered to help determine dose and dosing intervals.
- The characteristics of trial subjects needed for achieving the goal of the study.
- The time required and project management skills needed to set up a study, identify, assess and recruit investigators and gain their cooperation.
- The time and resources required to perform all tasks in a clinical trial.
- The role of CROs (Contract Research Organisations) in the conduct of clinical trials.
- The legal and ethical factors impacting clearance for clinical trial supplies, Case Report Forms (CRFs) and other relevant materials.
- The practicalities essential to the conduct of clinical trials at the research site.
- The ICH Good Clinical Practice (GCP) principles and practices and their application throughout the development programme.
- The content and purposes of the Trial Master File and the Investigator Site Files.

## Skills:

- To write a reasoned critique on whether there are appropriate safety data to proceed into clinical efficacy trials for a new drug.
- To evaluate the clinical pharmacology data for a new drug.
- To recommend and discuss the required characteristics of patients within the CDP.
- To prepare and execute a feasibility plan.
- To contribute to the development of a project management plan for the clinical development of a new product. This should include key milestones.
- To describe how to arrange appropriate legal and ethical clearance for clinical trial supplies, Case Report Forms (CRFs) and other relevant materials.
- To prepare a plan of human and financial resources.
- To prepare a risk-adapted monitoring plan.

## Behaviours:

- Recognises that there are many non-pharmacological aspects that determine whether a drug can proceed to efficacy trials e.g. formulation issues, cost of goods.
- Consults with colleagues in the product development team on the impact of early development data on the direction and design of Phase III confirmatory studies.
- Consult with medical experts on the availability of patients and the effects of interfering therapies on the trial conduct.
- Recognises that successful product development requires a multi-disciplinary team approach to which the pharmaceutical physician / MDS must make timely and effective contributions.
- Recognises the need to be extra vigilant when studies are planned for countries where ethical and legal standards are more difficult to enforce.
- Consults with colleagues in the product development team on the impact of delays to the project plan and how these may be minimised or compensated.
- Consults with the investigators the need and requirements of adequate quality management.
- Recognises the need for stringent adherence to procedures and maintenance of full and accurate records.
- Ensures that quality assurance and quality control tools are available for effective implementation.

## C11. THE DESIGN AND EXECUTION OF CONFIRMATORY STUDIES AND EVALUATION OF THE RESULTING DATA AS APPLIED TO THE CDP AND THE TARGET PRODUCT PROFILE (TPP).

**Applied knowledge of:**

- The research, studies and data that should be available to make an informed decision to proceed to clinical confirmatory studies (Phase III).
- The types of adverse effects that are likely to be encountered in clinical trials for the pharmaceutical agent under development.

## Skills:

- To write a reasoned critique on whether there are appropriate efficacy and safety data to proceed into clinical confirmatory trials for a new drug (real or hypothetical).
- To choose the most appropriate study design for a confirmatory trial.
- To evaluate the results of a clinical study in the context of the TPP.

## Behaviours:

- Recognises the importance of a medical input to the evaluation of early exploratory development data, and shares this with the product development team.
- Recognises the value of healthy volunteer and human tolerability studies in product development and participates actively in their evaluation.
- Consults with colleagues in the product development team on the impact of early development data on the direction and design of confirmatory studies.

## C12. EVALUATION AND INTERPRETATION OF THE PRINCIPLES FOR THE DEVELOPMENT OF A CLINICAL TRIAL PROTOCOL APPLYING PRINCIPLES OF GCP AND CLINICAL PHARMACOLOGY

**Applied Knowledge of:**

- The ICH Good Clinical Practice (GCP) principles and practices and their application throughout the development programme.
- Safeguards for volunteer participants in early-phase studies and those for patients that must be followed.
- The required critical data in a study.

## Skills:

- To plan or review a series of clinical pharmacology investigations, within the GCP framework, in a sensible stepwise sequence in order to characterise the compound’s properties and to allow critical judgements to be made on its therapeutic potential and safety.
- To ensure that quality assurance checks are made and acted upon.
- To contribute to the design and preparation of a study protocol for a new drug
- To review outline protocols considering:

-How they achieve the aims of the Clinical Development Plan (CDP).

-How they comply with ethical requirements.

## Behaviours:

- Ensures that as a team member the welfare of subjects in clinical studies, whether therapeutic or non- therapeutic, is paramount.
- Treats all trial subjects as individuals with courtesy, empathy, compassion, professionalism and respect for their dignity.
- Recognises the need for stringent adherence to procedures and maintenance of full and accurate records.
- Recognises the value of carefully thought out protocols in clinical development and participates actively in their development.
- Is aware of how clinical trials can be conducted in the real world within the target timelines and allocated budget and the need for a risk management plan for the project.

**C13. SUMMARY OF THE PRINCIPLES OF CASE REPORT FORM DESIGN AND CLINICAL DATA**

## MANAGEMENT, INCLUDING CDISC, EDC AND MedDRA.

**Applied Knowledge Of:**

- The key areas where data management contributes to the clinical trial process.
- The design of Case Report Forms (CRFs) and other data capture tools, both electronic and paper-based, and their key features to ensure that data are collected in a practical and unambiguous way.
- The impact of poor CRF design on the conduct of the clinical trial.
- Common issues with CRF completion at the study site and the importance of training site staff and CRAs (Clinical Research Associates).
- The data cleaning process (relevant to both electronic and paper CRF) and where the data validation plan should be reviewed.

## Skills:

- To list the key areas where data management contributes to the clinical trial process.
- To identify examples of good CRF design practice and examples of poor CRF design.
- To identify common problem areas in CRF completion.
- To describe the data cleaning process post data-entry in a paper environment.
- To describe the typical contents of a Data Validation Plan.
- To list examples of areas where consultative advice to resolve data issues can be provided.

## Behaviours:

- Recognises the need to involve data management in all phases of a clinical trial to ensure efficient data collection and robust data for analysis.
- Recognises the importance of pharmaceutical physicians / MDS and clinical investigators contributing to reviews of CRF design.
- Recognises the need for data management to be involved in training site staff and CRAs during study set- up.
- Recognises the importance of pharmaceutical physicians / MDS participating in data review, and providing advice to teams, to ensure a clinically correct database for analysis.

**C14: THE ACTIVITIES AND PROCESSES IN THE SELECTION AND MANAGEMENT OF SITES FOR**

## CLINICAL TRIALS.

**Applied Knowledge of:**

- The time required and project management skills needed to set a study.
- The role of CROs (Contract Research Organisations) in the site management support conduct of clinical trials.
- The legal and ethical factors impacting clearance for clinical trial supplies, Case Report Forms (CRFs) and other relevant materials.
- The elements of Investigator contracts, hospital/institution contracts, patient liability insurance, co- investigator contracts, publication rights
- The content and processes of financial management of a clinical trial, especially in a multi-national setting
- The requirement of professional handling and managing investigational drug supply
- The requirements for: financial disclosure and data protection.
- SOPs, Audit and inspection procedures applied to studies before, during and after their conduct.
- The reasons and elements of for internal QA procedures and the possibilities for mandated external audits and inspections.
- The role and responsibilities of the QA department.
- The role and content of training in clinical trials

## Skills:

- To use principles of resource management and financial management to prepare a Clinical Trial Management Plan.
- To prepare SOPs, Work Instructions and related forms.
- To ensure reliable quality management in all aspects and define the criteria of the selection of clinical trial sites.
- To prepare sites for particular studies regarding number and execute a Training Plan.
- To oversee the regional site distribution in order to ensure efficiencies in time, quality and costs.
- To assess the time required and project management skills needed for a site to set up a study.
- To identify, assess, recruit and manage investigators / site staff.
- To utilise the tools for and understand the complexity of study participant recruitment planning and gain the site cooperation.

## Behaviours

- Is aware of the critical importance of the selection of sites of clinical trials taking into account quality, time and costs.
- Recognises the role of pharmacists in the management and quality control of investigational drug supply
- Recognises the need to be extra vigilant when studies are planned for countries where ethical and legal standards are more difficult to enforce.
- Recognises that successful product development requires a multi-disciplinary team approach to which the pharmaceutical physician / MDS must make timely and effective contributions.
- Consults with colleagues in the product development team on the impact of delays to the project plan and how these may be minimised or compensated.

**C15. PROVISION OF THE CLINICAL INPUT INTO THE DESIGN AND REVIEW OF A STATISTICAL**

## ANALYSIS PLAN.

**Applied Knowledge of:**

- The structure of a Statistical Analysis Plan.
- The role of hypothesis testing, P-values, parametric and non parametric analysis, summary statistics, confidence intervals and modelling in the statistical analysis of data.
- The methods of statistical analysis for investigating the homogeneity of the treatment effect.
- The methods of statistical analysis for the detection of fraud and misconduct.
- The thinking behind statistical methods, such as the pre-specification of methods of analysis, the control of type I error, the concept of statistical power and its application in sample size estimation and the principle of intention-to-treat to reduce bias.
- The concepts of sensitivity and specificity in diagnosis.
- Statistical methods, such as the analysis of covariance and the choice of statistical test, for maximising precision when analysing data.
- The methods of statistical analysis for investigating the homogeneity of the treatment effect.
- The methods of statistical analysis for the detection of fraud and misconduct.

## Skills:

- To review a Statistical Analysis Plan.
- To document the reasons for the inclusion/exclusion of patients.
- To identify the reasons for the inclusion/exclusion of patients in samples for analysis.
- To plan the presentation of the results of the statistical analysis in the clinical study report.
- To interpret the results of a statistical analysis of data based on methods including: survival analysis; analysis of covariance; logistic regression; meta-analysis.

## Behaviours:

- Recognises the role of the Pharmaceutical Physician / Medicine Development Scientist in the construction and review of a Statistical Analysis Plan.
- Recognises the importance of the use of appropriate statistical methodology for the correct interpretation of clinical studies.

**C16. APPRAISAL AND REVIEW OF RELEVANT LITERATURE AND OTHER SOURCES IN**

## PREPARATION OF MANUSCRIPTS FOR PUBLICATION.

**Applied knowledge of:**

- Literature in the field and of important requirements to meet clinical needs.
- Relevant statistical methods and analyses.
- Pharmacokinetic analyses and modelling.
- The key statistical aspects of a clinical study that should be included in a publication or study report.
- The differences in the structure and content of documentation for a clinical study report and a publication.
- The transparency requirements for result reporting.
- The establishment and use of clinical trial registries.
- The process involved in the preparation of clinical study reports and manuscripts reporting clinical studies for submission for publication to a peer-reviewed journal, the key issues which must be addressed, and the typical structure of such a manuscript.
- To interpret and explain the results of clinical studies.
- To explain the relevance of meta-analysis for medical decisions.

## Skills:

- To conduct or review a literature search concerning clinical pharmacology and research methodology.
- To review and discuss all relevant publications.
- To provide a comprehensive review of a therapeutic field and the met and unmet needs.
- To summarise the results of a programme of clinical research.
- To assess the design and conduct of studies for a product (real or hypothetical).
- To review the results to determine the clinical significance of the data.
- To assess the risks and benefits of a potential new medicine.
- To explain the relevance of meta-analysis for medical decisions.
- To initiate and update clinical trial information and result reports in all applicable databases.
- To explain how Phase 0 and I fits into an overall Clinical Development Plan (CDP).
- To review or contribute to the preparation of a manuscript or document as a joint author on clinical studies for submission to a peer-reviewed journal or a regulatory authority.
- To interpret the results of a statistical analysis based on commonly used statistical procedures presented in a publication or report.
- To write clear, coherent and comprehensive reports of clinical research undertaken.
- To summarise the results of a programme of clinical research.
- To assess the design and conduct of studies for a product.
- To review the results to determine the clinical significance of the data.

## Behaviours:

- Maintains knowledge of current literature in the relevant therapeutic field and familiarity with recent advances in clinical pharmacology and therapeutics and its impact on the pharmaceutical business.
- Recognises the need to be able to review published clinical studies and the importance in the publication of a full description of statistical methodology.
- Recognises the time-limitation of publications in a rapidly developing technical field.
- Recognises the scientific and ethical imperative to submit the results of scientific research to peer review.

**C17. INTERPRETATION OF AND EXPLANATION FOR THE OUTCOME OF CLINICAL STUDIES.**

## Applied knowledge of:

- The main imaging techniques used currently in clinical trials.
- The main laboratory methods used in clinical trials.
- The principles of Good Laboratory Practice (GLP).
- The use and examples of surrogate markers.
- The process involved in the preparation of clinical study reports and manuscripts reporting clinical studies for submission for publication to a peer-reviewed journal, the key issues which must be addressed, and the typical structure of such a manuscript.
- Patient outcomes endpoints.

## Skills:

- To interpret the results of a statistical analysis of data based on methods including: survival analysis; analysis of covariance; logistic regression; meta-analysis.
- To recommend, with reasons, appropriate imaging, laboratory methods and surrogate markers for a study protocol.
- To interpret and explain the results of clinical studies.
- To recommend and/or oversee site inspections & audits related to the study report and make personal visits to sites as required.
- To write clear, coherent and comprehensive reports of clinical research undertaken.
- To summarise the results of a programme of clinical research.
- To assess the design and conduct of studies for a product.
- To review the results to determine the clinical significance of the data.
- To assess the risks and benefits of a potential new medicine.
- To explain the principles of meta-analysis.

## Behaviours:

- Recognises the contribution of new technologies and approaches in the field of clinical research on new medicines.
- Communicates advances in clinical pharmacology and clinical practice.
- Recognises the importance of the use of appropriate statistical methodology for the correct interpretation of clinical studies.
- Recognises the need to interpret and disseminate clinical research data within the team, company and scientific community via peer-reviewed publication in a timely and effective manner.
- Recognises the impact of clinical trial data on the stock market.

**AREA 2**

**REGULATORY AFFAIRS AND SAFETY OF MEDICINES**

# Domain 3: Medicines Regulation

## The Pharmaceutical Physician / Medicines Development Scientist is able to interpret effectively the regulatory requirements for the clinical development of a new drug through the product lifecycle to ensure its appropriate therapeutic use and proper risk management.

**C18. SUMMARISES THE LEGISLATIVE FRAMEWORK SUPPORTING THE DEVELOPMENT AND**

**REGISTRATION OF MEDICINES , ENSURING THEIR EFFICACY, SAFETY AND QUALITY.**

**Applied Knowledge of:**

- National and Regional Regulations, Directives and Guidelines relating to medicines’ development and monitoring of safety and quality (FDA, EMA, WHO, PMDA, others and contents related to product information.
- The ICH (International Conference on Harmonisation) process and the significance of ICH Guidelines.
- The requirements for development and registration of medicines.
- Regulations, Guidelines, Formats and Contents relating to writing Product Information such as:
  - Summary of Product Characteristics (SmPC) or equivalents.
  - Product labels or Package Inserts; - Patient Information Leaflets (PILs).
  - Technical Leaflets; - Package Labelling; - Advertisements.
- Data requirements supporting the contents of these documents. Penalties for breaches.
- The statutory sights under which Regulatory Agencies perform Inspections.
- Country/Regional requirements for medicines development in the context of the ICH guidelines and their implementation.
- Confidentiality agreements and other interactions between regulatory agencies worldwide.
- Interactions between companies and regulatory agencies: US and EU.

## Skills:

- To identify, retrieve and assemble documents from all available sources in order to be informed about and to undertake specified regulatory tasks.
- To discuss the distinction between enforcement and implementation of Laws, Regulations, Directives and Guidelines.
- To describe the relationship between regional and national regulatory authorities.
- To work with Agencies and other groups as required with regard to legal and regulatory requirements.
- To describe the role and responsibility of the Qualified Person for Pharmacovigilance (QPPV).
- To describe format and content of Product Information documents.
- To write and / or review Product Information documents.
- To review product-related literature to ensure it is consistent with the terms of the Product Information (SmPC, PIL) and any company core documents, and complies with appropriate regulations and codes of practice.
- To describe the circumstances under which Regulatory Agencies may order an inspection and be able to address questions raised by the Inspectorate.
- To understand the collaboration, communication and exchange of information between regulatory agencies on pertinent matters of drug development.

## Behaviours:

- Recognises the need to maintain close contact and work collaboratively with the Regulatory Affairs Department and the Market Access Team.
- Promotes informed reflection on legal and regulatory issues within a team.
- Recognises the importance of working with regulatory colleagues and including, where appropriate, those in the national agencies, to advance medicines development and patient safety.
- Seeks advice from experts and participates in appropriate multidisciplinary meetings to ensure the safety, efficacy and quality of medicines.
- Recognises the Product Information documents (SmPC, PIL) or equivalents to be legally binding documents that impact the contents of other product-related literature.
- Understands the need to regularly scrutinise the documents relating to Product Information (SmPCs, PILs) to ensure their accuracy and effectiveness in promoting the safe and effective use of medicine.
- Recognises the importance of inspections and is well-prepared for them, ensuring that records are complete and up to date.
- Appraises the impact of collaboration between regulatory agencies including joint procedures offered to pharmaceutical companies.

**C19. THE REGULATIONS RELATED TO POST-AUTHORISATION SAFETY MONITORING AND**

## REPORTING PROCEDURES.

**Applied Knowledge of:**

- The differences among national/regional Adverse Drug Reaction (ADR) reporting systems, including spontaneous reporting schemes.
- Drug safety reporting requirements that operate under different legal jurisdictions.
- Pharmacovigilance requirements and activities at national, regional and international levels.
- How to submit regulatory reports for:
  - marketed products;
  - clinical trials concerning marketed products;
  - registration dossiers for products already marketed elsewhere.
- The obligations of the Marketing Authorisation Holder (MAH) with respect to product safety reporting.
- The strengths and limitations of various kinds of reports and approaches to identifying safety issues.
- Methods allowing for the further investigation and assessment of product safety alerts.
- The underlying principles for risk minimisation strategies.
- The role and conduct of post-authorisation safety studies (PASS).

## Skills:

- To identify safety signals that are likely to be of greatest concern to regulatory agencies and the impact these may have on the future development or use of the product.
- To apply the actions required for the reporting of safety signals and anticipate the immediate and / or future impact of those signals on the development of a product.
- To participate in internal company meetings and, where required, advise on dialogue with the regulatory agencies to discuss post-authorisation safety signals, reports and potential studies to investigate safety.
- To identify various sources of safety data required in an assessment of a (real or hypothetical) safety issue and review / prepare a report for submission to a regulatory authority.
- To work in consultation with experts and regulators to evaluate new signals.
- To discuss the regulatory approaches to the investigation and assessment of product safety issues and dissemination of information to regulators, investigators and clinicians.
- To advise on the requirements regarding the timelines for submission of safety reports to regulatory agencies.
- To devise an outline for or appraise a risk management plan (RMP) for a product (real or hypothetical).

## Behaviours:

- Recognises the necessity for monitoring the safety of marketed products and having effective adverse drug reaction reporting systems.
- Recognises the need for Pharmaceutical Physicians / MDS to maintain close collaboration with both the product safety department and regulatory agencies.
- Recognises the strengths and limitations of in-house systems for collating and interpreting safety databases.

**C20. EXPLANATION FOR THE SIGNIFICANCE OF REGULAR PRODUCT SAFETY UPDATE REPORTS**

## TO THE REGULATORY AGENCIES AND PARTICIPATE IN THEIR PREPARATION AND REVIEW.

**Applied Knowledge of:**

- CIOMS and the history of required Safety Update Reports; particularly Periodic Safety Update Reports (PSURs) and Annual Safety Reports (ASRs) / Development Safety Update Reports (DSURs).
- The legal requirements for Safety Update Reports including contents, format and periodicity.
- The possible outcomes from the review of a Safety Update Report.
- The differences between the various types of Safety Update Reports.

## Skills:

- To describe the differences between the various types of Safety Update Reports.
- To review and assess the known safety information for inclusion in reports.
- To assess the content of Safety Update Reports in the context of current prescribing information for a product and the implications for patient treatment / prescribing practice.
- To contribute to an update of prescribing information to promote the safe and effective use of medicines

## Behaviours:

- Recognises that Safety Update Reports are a vital means of reviewing proactively the safety of marketed products and products in development.
- Realises that Safety Update Reports enable different authorities to be provided with the same comprehensive information on the safety of the drug from all sources.

**C21. EVALUATION OF THE UNLICENSED USE OF MEDICINES AND ENSURING PATIENT SAFETY.**

## Applied Knowledge of:

- Legislation that allows for provision of unlicensed medicines for specific uses.
- The types of unlicensed use, including compassionate use / named patient supplies.
- Differences between off-label and unlicensed medicines.
- Sources of unlicensed medicines and impact of Internet advertising and pharmacies.
- Procedures for gaining approval for the provision of unlicensed medicines to healthcare professionals.
- Safety monitoring requirements and procedures during unlicensed use of medicines.
- Measures to promote the use of medicines as approved and avoid the unauthorised use of unlicensed medicines.
- Penalties for promoting the off-label use of medicines.

## Skills:

- To differentiate between the use of medicines when off-label and when unlicensed, and between the various types of unlicensed medicines.
- To discuss conditions placed on the use of various types of unlicensed medicines to protect patients.
- To review and assess requests for making unlicensed medicines available to select groups.
- To generate scientific arguments supporting the availability of medicines for unlicensed use.
- To discuss the duties of the supplier and the prescriber with respect to unlicensed medicines.
- To discuss procedures for gaining approval for provision of unlicensed medicines.
- To review applications for making available unlicensed medicines and liaise with regulators.
- To discuss the risks of Internet advertising and procurement of medicines.
- To discuss how to avoid unauthorised use of unlicensed medicines.
- To appraise requests for making available unlicensed medicines and to offer advice on how to deal with these requests.

## Behaviours

- Recognises why unlicensed product use may be necessary.
- Understands the need to consult and discuss with regulatory colleagues when requested to make unlicensed product available.
- Is able to balance objectively and illustrate the risks and benefits of making available unlicensed medicines.
- Takes a responsible approach to balancing the risks and benefits regarding the availability and use of unlicensed medicines.
- Demonstrates an understanding of country-specific provisions with respect to the supply of unlicensed medicines.

**C22. PROCEDURES IN THE DEVELOPMENT AND RENEWAL OF MARKETING AUTHORISATIONS**

## Applied knowledge of:

- The structure of the Common Technical Document (CTD).
- The contents of a registration dossier.
- National procedures.
- European centralised, decentralised and mutual recognition procedures for Marketing Authorisation.
- Use of clinical development guidelines and the value and use of scientific advice from national and international Regulatory Agencies.
- Scientific advice procedures.
- The role of advisory bodies.
- The structure and contents of a Clinical Overview.
- Procedures in ICH regions.
- Appeal and arbitration procedures.
- International differences in the requirements for Marketing Authorisation renewal.
- Relevant regulations: Orphan drugs and Paediatrics.
- Biosimilars - Regulatory background, practical challenges during development (e.g. CMC, extrapolation of clinical data), interchangeability, nomenclature, uptake in medical practice.

## Skills:

- To discuss regulatory evaluation and approval processes.
- To understand the rationale behind the CTD and its relationship to data normally required for registration.
- To differentiate between the differences between the ICH regions and discuss their advantages and disadvantages.
- To discuss the value and timing of scientific advice.
- To write and / or review a briefing package when seeking scientific advice from a regulatory body and participate effectively during the scientific advice meetings or in later meetings to discuss advice received.
- To discuss the data requirements for different types of post-approval applications.
- To discuss the procedures for extending an indication or target population and amending dose schedules and safety information.
- To appraise the applicability and regulatory requirements of different formulations and product combinations.
- To write or review the clinical section of a CTA and / or an IND.
- To write and / or appraise Clinical Overviews for a new drug application (or variations, line extensions, abridged documents).
- To address questions, particularly on clinical data or content of proposed product information documents raised during the Regulatory Agency review of new drug applications.
- To discuss the concept, value and timing of orphan drug designations.
- To write and/or review a Paediatric Investigations Plan (PIP) or Pediatric Study Plan (PSP).
- To evaluate the specific requirements for the development and licensing of biosimilars.

## Behaviours:

- Appraises the advantages and disadvantages of different procedures with regard to specific therapeutic classes of drugs.
- Recognises the advantages and disadvantages of scientific advice.
- Recognises the significance of a well-written Clinical Overview.
- Recognises that a Marketing Authorisation is an evolving document and the role of medical and regulatory professionals in its evolution.
- Recognises the need to monitor the opportunities and threats to a marketed medicine.
- Recognises their legal obligation to protect public safety and promote the safe and effective use of medicinal products.
- Recognises the advantages and disadvantages of orphan drug designations.
- Recognises the significance of compliance with paediatric regulatory requirements (PIP, PSP).
- Demonstrates an understanding of the unique regulatory requirements of biosimilars and how to address them in the development plans.

**C23. THE DESIGN, PREPARATION, REVIEW AND EVALUATION OF CLINICAL OVERVIEWS FOR**

## REGULATORY SUBMISSION.

**Applied Knowledge of:**

- The structure and contents of a Clinical Overview.

## Skills:

- To write and / or appraise Clinical Overviews for a new drug application (or variations, line extensions, abridged documents).
- To address questions, particularly on clinical data or content of product information documents raised during Regulatory Agency review of new drug applications.

## Behaviours:

- Appraises the advantages and disadvantages of different procedures with regard to specific therapeutic classes of drugs.
- Recognises the advantages and disadvantages of scientific advice.
- Recognises the significance of a well-written Clinical Overview.

**C24. THE LEGAL FRAMEWORK FOR CLINICAL TRIALS AND THE REQUIREMENTS IN DIFFERENT**

## REGIONS, AND PERCEIVED PROBLEMS ASSOCIATED WITH GLOBAL DRUG DEVELOPMENT.

**Applied Knowledge of:**

- The national and international laws, regulations and guidance relating to clinical trials.
- National guidance relating to First-in-Human (FIH) studies and the legal obligations of centres conducting FIH studies.
- The contents of the Investigators’ Brochure (IB or IDB).
- Clinical Trials Applications (CTAs).
- National clinical trials legislation including conditions placed on study amendments, ongoing safety reports and termination.
- ICH Good Clinical Practice (GCP) and its impact upon the validity of a licence application.
- Investigational New Drug (IND) procedures in ICH regions.
- Problems associated with global drug development.

## Skills:

- To discuss the impact and implications of the laws, regulations and guidance relating to clinical trials on the establishment of clinical trials and drug development programmes.
- To write medical contributions to and / or review the IB/IDB
- To discuss the obligations of the sponsors of clinical trials.
- To contribute to the writing of protocols for clinical trials.

## Behaviours:

- Recognises the need to regulate clinical trials.
- Adopts a highly ethical and scientific approach to setting up clinical trials.
- Recognises that investigators and regulators need up-to-date safety information for safe conduct and appraisal of clinical trials.
- Recognises the risks (ethical, clinical and regulatory) associated with poorly designed or executed clinical trials.
- Recognises the importance of close collaboration with investigators and Regulatory Authorities on the progress of clinical trials.

**C25: THE MECHANISMS FOR WIDER AVAILABILITY OF MEDICINES, AND CONTRIBUTION TO**

## PRODUCT DEREGULATION.

**Applied Knowledge of:**

- The regulatory procedures for changing the legal classification of medicines.
- Local national classification systems for availability of medicines
- Patient Group Directions.
- Controls over the use and promotion of non-prescription medicines.
- Monitoring the safety of non-prescription medicines.
- Specialised regulatory procedures for expedited drug development (e.g. Fast Track, Breakthrough therapy designations, Accelerated assessment, PRIME, Adaptive Pathway etc.)
- Parallel scientific advice regulatory agencies/HTA bodies.

## Skills:

- To evaluate and advise on data in relation to proposed changes in legal classification.
- To assess the effect of changes in legal classification on:
  - public safety;
  - public health.
- To review and / or produce an outline of a Clinical Overview for a legal status reclassification application.
- To evaluate the safety implications of making drugs available over-the-counter.
- To discuss the limitations placed on the advertising of non-prescription medicines.
- To discuss the value of various specialised (optional) regulatory procedures and to appraise a programme’s eligibility.
- To discuss the value and timing of scientific advice, including the option of jointly engaging with regulatory agencies and HTA bodies.

## Behaviours:

- Recognises the advantages and potential disadvantages of the deregulation of medicines.
- Understands the need to consult relevant stakeholders when considering the deregulation of a medicine.
- Understands the importance of monitoring the safety of medicines available without a prescription.
- Ensures that promotion of the product to the public is responsible.
- Recognises the advantages and disadvantages of specialized regulatory procedures for expedited drug development.
- Recognises the advantages and disadvantages of scientific advice, including parallel scientific advice from regulatory agencies/HTA bodies.

## C26: THE ORGANISATION OF THE INVESTIGATION OF PRODUCT DEFECTS, COUNTERFEIT PRODUCTS AND OTHER MISCELLANEOUS PHARMACEUTICAL PROCEDURES AND REQUIREMENTS.

**Applied Knowledge of:**

- Dealing with product defects.
- The investigation of product defects.
- Dealing with counterfeit medicines.
- The impact of product defects and counterfeit medicines on public health and safety.
- The maintenance of a manufacturer’s / wholesaler’s licence and the role and remit of inspection.
- The application for and maintenance of import and parallel import licences.
- Good Manufacturing Practice (GMP) and Good Laboratory Practice (GLP) as applicable to product defects and counterfeits.
- Good Distribution Practices (GDP).
- The regulation of other non-medicinal products e.g. herbals, cosmetics, medical devices.

## Skills:

- To evaluate reports of product defects and counterfeit medicines.
- To advise on the clinical significance of product defect and counterfeit medicine reports.
- To input into communications with regulators and customers relating to product defects and counterfeit medicines.

## Behaviours:

- Recognises the importance of appropriate management of product defects and counterfeit medicine reports in protecting patient safety.

**C27: THE PRINCIPLES AND PROCESS OF REGULATION OF MEDICAL DEVICES AND**

## BIOTECHNOLOGY FORMULATIONS.

**Applied Knowledge of:**

- The fields of medical devices, and biotechnology products and their distinguishing features related to technologies and industry.
- The background of medical device legislation: old approach; new approach (CE mark, mutual recognition principles, notified bodies, harmonised standards, freedom of circulation).
- The role of the regulatory agencies within ICH, notified bodies, manufacturer and accreditation bodies in the field of medical devices.
- The conformity assessment of medical devices based on essential requirements.
- The guidelines for development of medical devices.
- Standardisation and use of standards: regional and, national standards, ISO/IEC standards and harmonised standards.
- Classification and demarcation of products – with a basic knowledge of pharmaceuticals.
- The clinical development process with medical devices.
- The compilation of technical dossier/design dossier.
- Vigilance and risk management programmes for medical devices.
- The double pharmaceutical and technological requirements for the development of medicine-medical device combinations.
- The principles of using animal or human tissues as medical devices.
- The need and requirements for the joint development of targeted medicine therapy and companion diagnostics.
- The clinical significance of nano-technologies.

## Skills:

- To discuss regulatory evaluation and approval processes.
- To understand the rationale behind the CTD and its relationship to data required for registration.
- To discuss differences between the ICH regions and their advantages and disadvantages.
- To discuss the value and timing of scientific advice.
- To write and / or review a briefing package when seeking scientific advice from a regulatory body and participate during the scientific advice meetings or in later meetings to discuss advice received.
- To discuss the data requirements for different types of post-approval applications.
- To discuss the procedures for extending an indication or target population and amending dose schedules and safety information.
- To appraise the applicability and regulatory requirements of different formulations and product combinations.
- To write and / or appraise Clinical Overviews for a new drug application (or variations, line extensions, abridged documents).
- To address questions, particularly on clinical data or content of proposed product information documents raised during the Regulatory Agency review of new drug applications.

## Behaviours:

- Recognises the importance of these regulatory actions to help ensure patient safety.
- Recognises the risk of errors in medical devices represents to safety and the need to minimise the risk.
- Recognises the importance of evaluating and documenting quality, safety, and effectiveness of devices.
- Recognises the differences in product development and life cycle between drugs and devices.
- Recognises the need to monitor the opportunities and threats to a marketed device.
- Recognises the~~ir~~ legal obligation to protect public safety and promote the use of devices.

# Domain 4. Drug Safety Surveillance

## The Pharmaceutical Physician / Medicines Development Scientist is able to evaluate the choice, application and analysis of post-authorisation surveillance methods to meet the requirements of national / international agencies for proper information and risk minimisation to patients and clinical trial participants.

**C28. TO CONTRAST THE KEY REGULATORY REQUIREMENTS FOR PHARMACOVIGILANCE,**

**BOTH IN THE MAJOR ICH REGIONS AND LOCALLY, AND THEIR HISTORICAL BACKGROUND.**

**Applied Knowledge of:**

- The evolution of medicines safety surveillance methods, and pharmacovigilance regulations worldwide, their harmonisation, and inter- and intra-company reporting systems for assembling and reporting adverse events.
- The responsibilities and liabilities of investigators, clinicians, study monitors, sponsors and manufacturers in the pre- and post-marketing phases to detect, assess and report adverse events.
- The requirements and processes for reporting to the country/regional regulatory agency.
- The requirements for informing prescribers, investigators, ethics committees and regulatory agencies of important safety concerns.

The pharmacovigilance operations in the country/region and their requirements for reporting.

- The role and responsibilities of the Qualified Person in Pharmacovigilance (QPPV) or country equivalent.
- Relevant pharmacovigilance sections from international/regional Directives, Regulations and Guidelines (i.e. WHO, EMA, FDA, others).
- The major past ‘landmark’ safety issues with medicines.
- The CIOMS Working Groups and Reports.

## Skills:

- To communicate and discuss medicines safety regulations with colleagues.
- To locate sources of information on safety regulations and identify any new requirements, evaluating whether these have implications for revising processes to ensure compliance.
- To apply relevant guidelines and directives and ensure compliance with them in all aspects of pharmacovigilance.

## Behaviours:

- Is fully aware of the broader ethical, moral and professional responsibilities of Pharmaceutical Physicians/MDS with regard to drug safety.
- Recognises the importance of adherence to the regulations and the need to stay up-to-date with changes to regulations and guidelines.
- Recognises the importance of the QPPV role or its equivalent function at the country level.

**C29. CONDUCT OF THE ASSESSMENTS REQUIRED FOR DRUG SAFETY REPORTING BOTH AT THE**

## LEVEL OF THE INDIVIDUAL PATIENT (CASE REPORT) AND AGGREGATE REPORT.

**Applied Knowledge of:**

- The regulations relating to the collection and reporting of adverse effects relating to medicines in the country/region.
- The contents of aggregate safety reports required by regulatory authorities e.g. PSURs.
- The contents of safety sections of Patient Information Leaflets (PIL) and package information.
- Processes to collect analyse and report product quality complaints (PQCs) and any associated adverse events.

## Skills:

- To determine reporting requirements of an adverse event or suspected ADR.
- To review case studies in the current literature and case reports from clinical studies.
- To note information about benefit-risk including other suspected ADRs and transfer the information to the report formats for submission to regulatory agencies.
- To apply ethical judgements and ensure adherence to appropriate guidelines when carrying out post- marketing surveillance studies.
- To assess medically the serious adverse events (SAEs) from clinical trials and determine their causal relationship to the study drug and expectedness.
- To evaluate medically an existing PSUR (aggregated safety report).
- To write the overall safety evaluation section of a PSUR (real or simulated).
- To write and to be able to review the safety section of a PIL and package information.
- To evaluate the impact on patient safety and the relationship with patients, healthcare professionals and regulators of inadequately assessed and managed Product Quality Complaints (PQCs).
- To discuss potential mitigation activities for PQCs e.g. change to process, withdrawal of a batch.

## Behaviours:

- Recognises the importance of meeting the requirements for reporting of ADRs.
- Recognises the overall function of the PSUR in terms of updating any safety matters concerning a medicine.
- Recognises how important it is that the PIL or other similar documents are written with consideration for the target audience to aid compliance.
- Recognises the importance of identifying risks to product quality and managing these proactively.

**C30. SUMMARISES THE SPONTANEOUS REPORTING AND SIGNAL DETECTION**

## METHODOLOGIES AND MEDICAL ASSESSMENT OF ADVERSE EVENT/ADVERSE DRUG REACTION REPORTS AS PART OF CAUSALITY ASSESSMENT

**Applied Knowledge of:**

- The major pharmaco-epidemiological methods for approaching drug safety issues and the characteristics of the most commonly used databases.
- The major methods of post-marketing surveillance.
- The application of the requirements for Post-Authorisation Safety Studies (PASS).
- The common causal mechanisms for ADRs.
- The mechanisms of drug interactions.
- The principles of causality assessment and causality algorithms to classify events as to their likely causal attribution to a particular medicine.
- The characteristics that make an ADR reportable according to international guidelines.
- Definition of medication error, off label, overdose, misuse and pregnancy cases.
- The application of epidemiological methods to spontaneous reporting.
- Coding systems for drug safety e.g. MedDRA.
- The methods and applications of signal generation methods in pharmacovigilance and the processes required for prioritisation and evaluation of detected signals.

## Skills:

- To apply the definitions of adverse event, serious adverse event, unexpected / unlabelled adverse event, suspected adverse drug reaction and clinically significant abnormal laboratory test value; and discuss the differences between them.
- To assess adverse event / reaction reports and be able to evaluate the importance of temporal relationships, concomitant medications, pre-existing or concurrent illnesses and patient characteristics.
- To formulate appropriate follow-up questions to reporting healthcare professionals and consumers as well as specifying the data that are important in the assessment of adverse event / reaction reports.
- To evaluate some published research data.
- To assess medically the post-marketing suspected ADR reports and determine seriousness, causal relationship to suspect drug and expectedness.
- To assess ADRs and other relevant benefit-risk information reported in the literature.
- To assess potential signals by using appropriate methods to assess adverse event frequencies in an external adverse event database e.g. FDA AERS / WHO Uppsala.

## Behaviours:

- Recognises the need for clear definitions and procedures / guidelines for adverse event reporting by both healthcare professionals and consumers.
- Recognises the importance of signal evaluation, causality assessment and the communication of relevant findings as a key responsibility in helping to safeguard future patients.
- Recognises the importance of medical assessment of adverse events from all potential sources and its potential future use in treating or advising patients.

**C31. SUMMARISES THE PRINCIPLES AND METHODS OF EVALUATION OF RISK-BENEFIT**

## BALANCE AND THE PRINCIPLES AND METHODS FOR MANAGING RISK TO PATIENTS AND CLINICAL TRIAL PARTICIPANTS.

**Applied Knowledge of:**

- The principles and methods for risk / benefit evaluation and related decisions during medicines development.
- The principles and process for development of safety specifications documents.
- The CIOMS VI report in respect of safety in clinical trials.
- The structure, roles and responsibilities of data safety monitoring committees.
- The principles of risk and benefit assessment based on the report of CIOMS IV Working Group.
- The principles and methods of post-marketing risk management plans (based on ICH E2E).
- The options and mechanisms for optimising safety in relation to benefit.
- Risk management issues relevant to pharmaceutical medicine; sources of risk, risk management tools, techniques and protocols.

## Skills:

- To review the relevant documents e.g. protocol, PIL, safety specifications, risk management plan, for appropriate risk and benefit statements.
- To make appropriate medical contributions for effective risk management plans by identifying risks to patients, potential risks and missing information and proposing appropriate risk mitigation activities.

## Behaviours:

- Recognises the contribution to be made by the pharmaceutical physician / MDS in risk / benefit assessment.

**C32. DISCRIMINATE AND EXPLAIN THE VARIETY OF REGULATORY ACTIONS POSSIBLE TO**

## ADDRESS CONCERNS ABOUT PATIENT SAFETY.

**Applied Knowledge of:**

- The key regulatory actions including Marketing Authorisation (MA) variations, urgent safety restrictions, MA suspension and withdrawal.
- The country/regional/international procedures for reassessment of risk- benefit balance of a medicine.
- The occurrence of medication error within a clinical setting and the potential consequences of use outside the labelled recommendations.
- Historical examples of medication error e.g. intrathecal administration of vincristine.
- The risk for a medication error occurring with a specific product and for including the identified risk in risk management documentation, risk mitigation plans and labelling with a specific product.

## Skills:

- To advise on the proper reaction to regulatory actions of the competent authorities e.g. MA variations, urgent safety restrictions, MA suspensions or withdrawals.
- To mitigate the risk of recognised sources of medical errors with a specific product (real or hypothetical).
- To review regulatory actions to show how each action can address concerns about patient safety.
- To evaluate the risk of medication error and propose measures for prevention (real or hypothetical).
- To identify sources of information on medication errors and the regulatory reporting requirements of identified cases.

## Behaviours:

- Recognises the importance of regulatory actions to help ensure patient safety.
- Recognises the risk that medication error represents to patient safety and the need to minimise the risk.

**C33. DESCRIPTION OF COMMUNICATION OF SAFETY ISSUES, THE VARIETY OF FORMATS**

## REQUIRED MEETING AUDIENCE NEEDS AND MEDICAL/SCIENTIFIC CONTRIBUTION TO THE DEVELOPMENT OF SUCH COMMUNICATIONS.

**Applied Knowledge of:**

- The possible regulatory actions available to address concerns about patient’ safety.
- The requirements regarding safety aspects of the Summary of Product Characteristics (SmPC or equivalent) and other information documents.
- Assessment of urgent safety issues, including product recall, and generation of appropriate communications to regulatory bodies, healthcare professionals and patients.
- The availability of urgent communication tools; the opportunities and pitfalls of their use.

## Skills:

- To review SmPCs or equivalent and other information documents on company products to ensure all safety issues are covered appropriately, completely and clearly.
- To evaluate and discuss urgent safety issues including patient communications and be able to write a Direct Healthcare Professional Communication (DHPC) letter for a real or hypothetical issue.
- To draft press briefings.
- To undertake communications planning and coordinate with key stakeholders the handling of a drug safety issue.

## Behaviours:

- Contributes to ensuring that the SmPC or equivalent reflects appropriately the safety profile of the medicine.
- Participates actively in the medical discussions around product recall.
- Shows an understanding of the needs of healthcare professionals in relation to urgent safety communications.
- Recognises the importance of communication of safety issues and the need for a variety of formats to meet different audience needs.

**C34. EVALUATION OF SAFETY ISSUES AND ESTABLISHMENT OF A CRISIS MANAGEMENT**

## TEAM, WITH RECOGNITION OF THE KEY FUNCTIONAL AREAS TO BE REPRESENTED AND THEIR ROLES AND RESPONSIBILITIES

**Applied Knowledge of:**

- The main steps involved in assessing and reacting to a potential crisis situation.
- The appropriate response to various simulated drug safety issues.
- The legal responsibilities and liabilities of pharmaceutical companies and Pharmaceutical Physicians / MDS in respect of drug safety issues.

## Skills:

- To define the organisation and conduct of a crisis management team for a real or hypothetical case.
- To identify the key individuals to be included in a crisis management team.
- To identify the main steps involved in assessing and reacting to a potential crisis situation.
- To respond appropriately to various simulated drug safety issues.

## Behaviours:

- Recognises the need for planned procedures to be in place and the urgency required to implement these plans appropriately.
- Consults effectively with all relevant parties.

**C35. APPRAISAL OF THE AREAS OF PROGRESS, LIKELY MAJOR ADVANCES AND CHALLENGES IN**

## DRUG SAFETY AND PHARMACOVIGILANCE.

**Applied Knowledge of:**

- - The role of clinical pharmacology, molecular biology, genomics and pharmacogenetics in the safety aspects of medicines development.
  - Safety aspects of Advanced-Therapy Medicinal Products (ATMPs).
  - Safety aspects of other innovative technologies.
  - The use of metadata in drug safety surveillance.
  - Challenges for pharmacovigilance, and evaluation of how any advances and developments may impact on medicines’ and patient safety surveillance.

## Skills:

- To identify the key markers and the methodology applied in order to demonstrate the efficiency of existing surveillance systems.
- To apply know-how of clinical pharmacology, genomics, pharmacogenetics and molecular biology to safety assessment in medicines development.
- To review the medical and scientific literature.
- To collect and collate data from multiple sources.
- To integrate and evaluate data and evidence from a safety issue or project.
- To prepare report(s) on safety issues and possible follow-on projects for internal use or for wider consumption and discussion.

## Behaviours:

- Demonstrates a willingness to remain abreast of advances in research and technology and apply new knowledge and learn new skills in the field of pharmacovigilance and risk management.

.

# Domain 5: Ethics and Subject Protection

## The Pharmaceutical Physician / Medicines Development Scientist is able to combine the principles of clinical research and business ethics for the conduct of clinical trials and commercial operations within the organisation.

**C36. EVALUATION OF THE IMPACT OF CULTURAL DIVERSITY AND THE NEED FOR**

**CULTURAL COMPETENCE IN MEDICINES DEVELOPMENT**

**Applied Knowledge of:**

- Professional codes of conduct and codes of ethical practice.
- Role of team dynamics in the way a group, team or department functions.
- Structures, roles and responsibilities of multi-disciplinary teams in pharmaceutical medicine / drug development.
- Techniques and methods for effective and empathic communication.
- Facilitation and conflict resolution methods.
- Leadership styles and approaches and applicability to different situations and people.
- Relevant legislation e.g. Equality and Diversity, Health and Safety, and local Human Resources policies.
- The duties, rights and responsibilities of an employer and of a co-worker.
- Organisational performance management techniques and processes.
- How healthcare governance influences patient care, research and educational activities at a local, regional and national level.
- The implications of change on health care systems and people.
- How decisions are made by individuals, teams and organisations.

## Skills:

- To take into account the social, ethical, economic and governmental factors in countries, which are local or regional and not necessarily international.
- To ensure that appropriate ethical review has been conducted and that a local independent ethical review is obtained when necessary.
- To communicate with team members with various educational backgrounds.
- To understand cultural and organisational differences in patient care and relations between patients and healthcare personnel.
- To organise efficient transfer and application of advanced healthcare technology to a different technical environment.
- To build training courses considering local educational background.
- To keep long-term follow-up running after introducing up to date technology to local healthcare.
- To use techniques and methods for effective and empathic communication.
- To use leadership styles and approaches with applicability to different situations and people.
- To use effective communications strategies.

## Behaviours:

- Avoids prejudice and preferences within self, others, society and cultures.
- Recognises the importance of ethnic, social, religious, public health and economic conditions prevailing in the country where clinical research is organised.
- Displays openness to different cultural behaviours.
- Considers the impact of different perspectives/ local prejudices in the local environment.
- Works and behaves in accordance with the local working habits within a project.
- Accepts the behaviours of differently socialised co-workers.
- Never comments negatively on local conditions and behaviours. If needed, initiates changes gradually and with respect.
- Uses local help to translate internationally accepted scientific or medical approaches to local environment.

**C37. ETHICAL ISSUES ASSOCIATED WITH CLINICAL RESEARCH, DRUG DEVELOPMENT AND**

## COMMERCIALISATION ON THE PRODUCTION OF SCIENTIFIC KNOWLEDGE.

**Applied Knowledge of:**

- Basic principles of the protection of research subjects and awareness of potential conflicts of interest.
- Practical procedures in providing full information to participants and their doctors, obtaining and recording their informed and continuing consent.
- Ethical review process from first in human to large scale clinical trials.
- Research methods and how to evaluate scientific publications.
- Impact of violation of intellectual property and confidentiality.
- Criteria for authorship, peer review process, for presentation or publication of research results.
- Need for data protection and transparency relating to the time of disclosing study data.

## Skills:

- To explain the conditions for involving patients in medicines development.
- To be conversant with the ethical principles and practices governing clinical research with participants.
- To explain how to avoid professional misconduct.
- To Identify and understand the consequences of plagiarism, misconduct and fraud.

## Behaviours:

- Recognises that clinical research requires the same and at times even greater scientific knowledge and medical responsibilities as those required in routine medical practice.
- Instils these principles and practices within the research organisation and local investigating teams.
- Recognises the ethical environment in which clinical trials are conducted and the contribution made by patients.
- Recognises that bias in patient selection because of race, gender, religious attitudes should be avoided.
- Involves local intermediaries when explaining the concept of the clinical trial and the ethical guiding principles. Local colleagues can ‘translate’ and explain the information according to local cultural understanding.

**C38. SIGNIFICANCE OF HISTORICAL ABUSES ON THE EVOLUTION OF PRINCIPLES OF HUMAN**

## SUBJECT PROTECTION.

**Applied Knowledge of:**

- The history of the development of ethical standards relating to biomedical research.
- Historical abuses of the evolution of principles of human subject protection and their significance.
- The investigators responsibilities for ensuring the safety and welfare of participants in clinical trials.

## Skills:

- To analyse the root causes, explain ethical problems and provide historical examples of abuses leading to the development of current laws and regulations.
- To analyse and address the ethical dimensions of any clinical research proposal.

## Behaviours:

- Be aware of the importance of the ethical principles of medical practice and clinical research as pertaining to rights and wellbeing of patients and research participants.
- Develop a behaviour showing acceptance of human dignity connecting people over cultural differences.

**C39. EVALUATION OF THE KEY DOCUMENTS RELATED TO THE ETHICAL CONDUCT OF CLINICAL**

## TRIALS.

**Applied Knowledge of:**

- The contents of the Declaration of Helsinki and Belmont Report and understanding of how the stakeholders view and apply these to clinical research.
- The ethical issues that might arise from clinical trials.
- The requirements of ICH Good Clinical Practice.
- The regulatory requirements for clinical trials, on country and regional bases.
- The Clinical Trial Application (CTA) procedures at the country and regional level.
- Process or ethical review by Ethics Committee and the national differences.
- The components of the informed consent process particularly in vulnerable populations according to the national requirements.

## Skills:

- To be involved in reviewing or writing and approving clinical study information sheets and consent forms for participants.
- To identify, understand and address ethical issues when reviewing a clinical trial protocol.
- To use appropriate lay language for study participants and their relatives whenever it is appropriate.
- To draft or review consent form and/or patient information sheet that meets all the national/regional requirements, in appropriate, patient-friendly language.
- To write or review a patient-centred ethical review application form.

## Behaviours:

- Recognises the inevitable ethical conflicts in clinical research (need for equipoise, placebo control, comparator treatment, follow-up treatment) and the necessity for balanced ethical judgement on matters of benefit and risk applied to the clinical development of medicines.
- Recognises and addresses the dilemma of the patient-physician relationship in standard healthcare and in clinical research.

**C40. DESCRIBES ETHICAL ISSUES INVOLVED WHEN DEALING WITH VULNERABLE**

## POPULATIONS, AND THE NEED FOR ADDITIONAL SAFEGUARDS.

**Applied Knowledge of:**

- The ethical issues and the international and local regulations and guidelines in particularly vulnerable patient groups (children, elderly, mentally incompetent, unconscious, socially or economically disadvantaged).
- The pros/cons of involving experts dealing with special vulnerable populations and local “lay community members” and/or patients into the ethical review process.

## Skills:

- To draft a clinical trial protocol or clinical development plan to be performed in a vulnerable patient group.
- To design an informed consent/assent process for patients unable to consent or with low literacy/cognitive impairment.

## Behaviours:

- Recognises the importance of paying special attention to the ethical problems pertinent for clinical research in vulnerable patient groups especially regarding informed consent and patient (study participant) selection.
- Acknowledges the need for additional expert advice for appropriate ethical review in studies in vulnerable populations, and when highly complicated trial designs and procedures are proposed.
- Is sensitive to the expectations, fears, concerns, burden and unsubstantiated hopes or other misconceptions within the local community when participating in clinical trials.
- Avoids raising unsubstantiated hopes and expectations within the local community when applying new high tech methods or new treatments.

**C41. COMPARISON OF THE REQUIREMENTS FOR HUMAN SUBJECT PROTECTION AND PRIVACY**

## UNDER DIFFERENT NATIONAL AND INTERNATIONAL REGULATIONS.

**Applied Knowledge of:**

- The sources and content of international and local regulations and guidelines for human subject protection and privacy.

## Skills:

- To apply national regulations in specific examples either virtually or real.
- To bridge the legislation and its local application and acceptance in terms of needs for transparency and patient confidentiality.
- To evaluate the local traditions and religion when a new legislation is accepted and particularly its introduction and acceptance by the community.
- To align the international requirements with the locally-accepted approaches to protect participants in clinical research and their privacy.

## Behaviours:

- Recognises the need to comply with national regulations governing protection of human subjects in clinical development.

**C42. ADOPTION OF THE PRINCIPLES OF CORPORATE SOCIAL RESPONSIBILITY**

## Applied Knowledge of:

- The process and potential liabilities when commissioning, funding and contracting.
- Relevant legislation e.g. Equality and Diversity, Health and Safety, Employment Law, and local human resources policies.
- The duties, rights and responsibilities of an employer and of a co-worker.
- Organisational performance management techniques and processes.
- How healthcare governance influences patient care, research and educational activities at a local, national and regional.
- Effective communications strategies within organisations.
- The transparency requirements by the national, regional and international regulations.
- Good Pharmaceutical Medical Practice e.g. Faculty of Pharmaceutical Medicine.

## Skills:

- To recognise, analyse and know how to deal with unprofessional behaviours.
- To create open and non-discriminatory professional working relationships with colleagues.
- To bring together different professionals and disciplines to provide high-quality multi-disciplinary teams.
- To develop effective working relationships with colleagues and others through good communication skills.
- To apply acquired knowledge and expertise in pharmaceutical medicine to assess and analyse situations, services and facilities in order to minimise risk to patients/participants and the public.
- To ensure the quality of equipment and safety of the environment relevant to the practice of pharmaceutical medicine.
- To apply acquired knowledge and expertise to pursue patients’ access to medicines.

## Behaviours:

- Adopts a patient-focused approach to decisions and acknowledges the rights, values and strengths of patients and the public.
- Recognises and respects diversity and differences in others.
- Accepts professional regulation.
- Recognises the need to promote professional attitudes and values.
- Recognises the need for probity and the willingness to be truthful and admit errors.
- Recognise the need for effective interaction with professionals in other disciplines and agencies.
- Respects the skills and contributions of colleagues.
- Will support the publication and communication of clinical trials results.
- Actively seeks advice when concerned about patient safety.
- Takes responsibility for performance, risk management and audit – in order to improve quality of services.
- Recognises the importance of best practice, transparency and consistency.
- Prevents and fights against bullying and harassment.

**AREA 3**

**HEALTH CARE AND PROFESSIONALISM**

# Domain 6: Healthcare Marketplace

## The Pharmaceutical Physician / Medicines Development Scientist is able to appraise the pharmaceutical business activities in the healthcare environment to ensure that they remain appropriate, ethical and legal to keep the welfare of patients and research participants at the forefront of decision making in the promotion of medicines and design of clinical trials.

**C43. DESCRIPTION OF THE COMMERCIAL HEALTHCARE ENVIRONMENT IN WHICH PHARMACEUTICAL MEDICINE OPERATES, IDENTIFYING THE CONTRIBUTION OF LAWS, AND OF REGULATORS AND OTHER STAKEHOLDERS IN THE DECISION-MAKING FOR PRESCRIBING MEDICINES.**

**Applied Knowledge of:**

- Components of the legal and regulatory framework in which pharmaceutical medicine operates (e.g. Advertising Regulations, Codes of Practice and Guidelines applying to national activities for the promotion of medicines – WHO, IFPMA, EFPIA, Local; National regulatory bodies; Good practices (GxPs); Product life-cycle management; Stakeholders and healthcare organisations (e.g. national/regional regulatory agencies).
- The contribution and decision-making processes, in relation to prescribing of:
  - The National Institute for Health and Clinical Excellence (NICE) or equivalent organisations aimed to assess cost effectiveness;
  - Value assessments of pharmaceuticals;
  - Disease management guidelines;
  - Drugs and Therapeutics Committees;
  - Computerised prescribing systems;
  - Reference works e.g. BNF, Martindale, MIMS, Drug Formularies.
- Distribution channels for medicines.
- The barriers and limitations to access to medicines in a specific market.
- Price/Cost Effectiveness.
- Principles of Health Economics.

## Skills:

- To analyse the roles, importance and relative contribution and interactions of organisations, laws and guidelines in supporting the legal and regulatory framework within which pharmaceutical medicine operates.
- To evaluate the major interactions between the key stakeholders in the healthcare marketplace.
- To interpret the interactions between the different groups and processes and how they can affect prescribing practices.

## Behaviours:

- Recognises the significance and authority of different levels of the law / regulation in the interpretation and operation of the legal and regulatory framework.
- Recognises how these groups influence prescribing practices within the healthcare environment.
- Recognises how these interactions influence the provision of healthcare.

**C44. THE KEY ELEMENTS INVOLVED IN MEDICAL-MARKETING COMMUNICATIONS IN THE**

## HEALTHCARE ENVIRONMENT AND EXPLANATION OF COMPLIANCE WITH REGULATION IN THIS CONTEXT.

**Applied Knowledge of:**

- The process involved in the preparation and production of legally compliant documentation to support medical-marketing activities.
- Product information legislation and guidance with reference to the national/regional pharmaceutical regulations.
- The structure and processes of the pharmaceutical industry internal environment.
- The pros and con of the various approaches to the structure of the medical function within pharmaceutical companies.
- Essential documents / SOPs required of a Company/medical organisation.
- Nature of organisational relations of Medical Department with other areas in the organisation.
- Principles of portfolio management.
- Financial aspect of the pharmaceutical operations, including return on investments.
- Power and Levels of authorisation (segregation of duties) of the medical organization.

## Skills:

- To assess the breadth of medical-marketing activities and materials, and to determine whether they are promotional and when and how they should be assessed for legal / regulatory compliance.
- To construct medical marketing materials / documents appropriate for the audience and consistent with the strategic direction for the promotion of the medicine. .
- To evaluate a range of medical marketing materials for scientific accuracy, legal and regulatory compliance and comprehension.
- To analyse selected materials and activities e.g. media communications, professional and public relations, pre-launch activities, with regard to scientific, educational and promotional content.
- To ensure that a balanced perspective (safety and efficacy) is evident in medicine promotion and communication.
- To be able to create alternative texts for advertising and promotion.
- To lead colleagues to a legally compliant and ethical position when debating on controversial promotional material.
- To guide and lead the debate on the public affairs and corporate relations issues as related to the medical marketing interaction in the healthcare marketplace.

## Behaviours:

- Recognises the importance and challenges of operating within a legal framework for medical-marketing communication and the consequences of non-compliance.
- Recognises the importance of ensuring the compliance of all product-related documentation with the content of the approved product information.
- Recognises the importance and consequences of differentiating medical communications from promotional materials and the need for such communications within the context of the business.
- Recognises how marketing research and profiling data can contribute to effective promotional activities while meeting the constraints of regulation in this context.
- Recognises the contribution and constraints of market research data in the promotion of medicines.

**C45. DESCRIPTION OF THE PHARMACEUTICAL INDUSTRY: INTERNAL ENVIRONMENT,**

## STRUCTURE AND FUNCTION, KEY STAKEHOLDERS AND COMMERCIAL DRIVERS AND EXPLANATION OF HOW THESE BUSINESS ELEMENTS IMPACT ON THE BROADER HEALTHCARE MARKETPLACE.

**Applied Knowledge of:**

- The process involved in the preparation and production of legally compliant documentation to support medical-marketing activities.
- Product information legislation and guidance with reference to the national/regional pharmaceutical regulations.
- The structure and processes of the pharmaceutical industry internal environment.
- The pros and con of the various approaches to the structure of the medical function within pharmaceutical companies.
- Essential documents / SOPs required of a Company/medical organisation.
- Nature of organisational relations of Medical Department with other areas in the organisation.
- Principles of portfolio management.
- Financial aspect of the pharmaceutical operations, including return on investments.
- Power and Levels of authorisation (segregation of duties) of the medical organisation.

## Skills:

- To analyse the impact of internal structures in the pharmaceutical industry on the interaction with the healthcare marketplace.
- To capitalise on the opportunities to perform effectively within the structures and functional organisation of the medical organisation.
- To target materials to the appropriate audiences e.g. journals / conferences / prescribers, and ensuring consistency with commercial messages.
- To assess the breadth of medical-marketing activities and materials, and to determine whether they are promotional and when and how they should be assessed for legal / regulatory compliance.
- To construct medical-marketing materials / documents appropriate for the audience and consistent with the strategic direction for the promotion of the medicine.
- To evaluate a range of medical marketing materials for scientific accuracy, legal and regulatory compliance and comprehension by the reader.
- To analyse selected materials and activities (e.g. media communications, professional and public relations, pre-launch activities), with regard to scientific, educational and promotional content.

## Behaviours:

- Recognises how internal business operations and drivers in the pharmaceutical industry impact the interactions with and relationships between the industry and the wider healthcare environment.

**C46. DESCRIPTION OF THE INFORMATION REQUIRED UNDERTAKING A COMMERCIAL**

## ANALYSIS OF THE MARKET POTENTIAL FOR A PHARMACEUTICAL PRODUCT/CANDIDATE WITHIN THE INDUSTRY BUSINESS ENVIRONMENT.

**Applied Knowledge of:**

- The elements involved in the commercial assessment of a pharmaceutical product e.g. profiling and positioning; clinical data pricing; products and services; intellectual property (IP); health economics, marketing related expenses.
- Generic and biosimilar medicines and their relevance within the healthcare system.
- The components and processes required for the evaluation of an in-licensing / collaboration option.

## Skills:

- To evaluate the commercial potential for a pharmaceutical product.
- To evaluate the commercial potential for an in-licensing opportunity for a pharmaceutical product.
- To identify the criteria for the inclusion of trials in a meta-analysis to answer key questions and present the results of such an analysis.
- To appraise a meta-analysis.
- To describe the statistical principles of benefit / risk assessments.
- To define appropriate parameters for database searches.
- To apply basic principles of health economics.

## Behaviours:

- Recognises the degree of data requirements and the inherent limitations in the commercial analysis of pharmaceutical product potential.
- Recognises the commercial potential and limitations of in-licensing and collaborative options.
- Recognises the importance of benefit / risk assessments in regulatory submissions.
- Recognises the importance of health economics in the development of submissions to review bodies.

**C47. APPRAISAL OF THE COMMERCIAL COMPETITOR ENVIRONMENT WHEN EVALUATING THE**

## OPPORTUNITY FOR A NEW MEDICINE UNDER DEVELOPMENT OR A CURRENTLY MARKETED PRODUCT.

**Applied Knowledge of:**

- The key components of a competitive commercial product analysis for a:
  - Marketed product;
  - Pipeline product;
  - Therapy area;
  - Competitor product.
- Health Technology Assessment (medicinal product) by the National / Regional Organisation
- (other) Approaches to health economic evaluation using cost-effectiveness and / or cost-minimisation.
- Demonstrate knowledge of assessing products in development and the feasibility of market access.

## Skills:

- To evaluate the commercial potential for a pharmaceutical product.
- To evaluate the commercial potential for an in-licensing opportunity for a pharmaceutical product.
- To perform a competitive product analysis for a product, at different stages in its development.
- To evaluate the promotional platform of a competitor product.

## Behaviours:

- Recognises the value of a robust competitive commercial product analysis for products at different stages in their development.

**C48. DESCRIPTION OF THE INTERFACE BETWEEN PHARMACEUTICALS AND THE EXTERNAL**

## STAKEHOLDER ENVIRONMENT, AND THE CHALLENGES BALANCING THE COMMERCIAL AND PROFESSIONAL ASPECTS IN MAKING ETHICAL JUDGEMENTS WITHIN THE LEGAL/REGULATORY FRAMEWORK.

**Applied Knowledge of:**

- The key stakeholders in the external environment the industry operates and how the industry’s activities impacts on them.
- The ethical issues related to the investigation and management of fraud and misconduct in sponsored clinical research.
- Creating a policy on information that may be supplied legally to patients (e.g. Data on File statement).
- Principles of corporate communications and maintenance of reputation.

## Skills:

- To evaluate the major interactions between the key stakeholders in the healthcare marketplace.
- To interpret the interactions between the different groups and processes and how they can affect prescribing practices.
- To perform an industry key stakeholder analysis.
- To perform balanced evaluations in the areas outlined.
- To give a balanced and objective clinical / scientific view / response in keeping with company strategy and professional ethics.

## Behaviours:

- Recognises the relevance of a stakeholder analysis and how it can contribute to the impact of developing better relationships and improving communication of the industry’s activities.
- Recognises the various approaches to business situations and the personal and professional challenges involved when making ethical judgements in the commercial environment.
- Recognises that all business decisions ultimately have to be seen for the consequences it may have for the patient.

# Domain 7: Communication and Management

## The Pharmaceutical Physician / Medicines Development Scientist is able to interpret the principles and practices of people management and leadership, using effective communication techniques and interpersonal skills to influence key stakeholders and achieve the scientific and business objectives*.*

**C49. DESCRIPTION OF THE PRINCIPLES AND PRACTICES OF PEOPLE MANAGEMENT AND**

**LEADERSHIP AND THEIR APPLICATION WITHIN THEIR OWN WORKING ENVIRONMENT; THE SETTING OF LEARNING AND IMPROVEMENT GOALS.**

**Applied Knowledge of:**

- Goal oriented selection of competent experts for the medical department.
- The components of employment legislation and company policies that are of relevance to the operation of a Medical Department and other areas of the pharmaceutical management environment.
- Roles and responsibilities of and relationships with key support functions e.g. Finance, Legal, Human Resources Departments.
- The elements of local Health and Safety legislation that have importance in the pharmaceutical environment.
- The principle of financing within the pharmaceutical sector as it applies to the industry, companies, departments and projects.
- The principles and practices of conducting competitive employment interviews and of selecting new staff.
- Methods of performance management and appraisal, their purpose, application and outcomes.
- The principles and common practices of managing a team (line or matrix).

## Skills:

- To identify regional or international differences between countries’ practices in the research-based pharmaceutical industry.
- To manage a budget and its accounts within a company, department or project.
- To differentiate between educational and performance appraisal.
- To describe the principles of effective objective setting.
- To describe sources of appropriate feedback on performance and their relative importance, how to give feedback, and measure outcomes.
- To apply motivational techniques in reaching a project outcome.
- To differentiate between training activities in medicines development and executive management training and how they can be complementary.
- To participate in succession planning and talent management initiatives.

## Behaviours:

- Recognises the importance of the legal framework of modern employment in the country/region and its impact on the work of drug development and commercialisation.
- Recognises the importance and impact of fiscal management in determining goals, priorities and outcomes in the pharmaceutical business environment.
- Recognises the importance of management / leadership style and influence on team dynamics and reaching departmental / project goals.
- Recognises the limits of own professional competence and practises accordingly.
- Recognises the importance of relationships and interactions between Medical and other key Departments

e.g. Marketing, Sales, R&D, Consumer Products (Over-the-Counter medicines).

**C50. THE COMMUNICATION OF KNOWLEDGE, SKILLS AND BEHAVIOURS ASSOCIATED WITH**

## THE COMPETENT PRACTICE OF PHARMACEUTICAL MEDICINE / MEDICINES DEVELOPMENT SCIENCE, USING THE BEST TECHNIQUES AND PRACTICES WHEN PARTICIPATING IN THE EDUCATION OF COLLEAGUES AND STAKEHOLDERS.

**Applied Knowledge of:**

- Relevant educational principles, notably in adult learning relevant to continuing education and professional development.
- The roles of bodies involved in medical and scientific education.
- The structure of an effective appraisal interview.
- Identification of learning methods and effective learning objectives and learning outcomes.
- The differences between formative and summative assessment and their role in an education and training programme.
- The differences between appraisal, assessment and performance review and their contribution to education, training and personal development.
- The role of workplace-based assessments, the selection, use and monitoring of assessment tools.
- The appropriate local course of action to assist a trainee experiencing difficulty in making progress within their training programme.

## Skills:

- To evaluate relevant educational literature.
- To adapt the teaching format according to the education situation and requirements of the audience, particularly non-medically qualified colleagues and staff.
- To provide effective and appropriate feedback after teaching and promote learner reflection.
- To conduct developmental conversations as appropriate, for example, appraisal, supervision, mentoring.
- To demonstrate effective lecture, presentation and small group teaching sessions.
- To lead or participate in departmental teaching programmes, including journal clubs.
- To identify and plan learning activities in the workplace.
- To manage personal time and resources effectively for the benefit of the educational programme and the needs of learners.

## Behaviours:

- Recognises the role of the pharmaceutical physician/MDS as an educator within the multi-disciplinary team.
- Recognises the need to incorporate educational opportunities within the workplace.
- Demonstrates willingness to teach trainees and others to maximise effective communication and practical skills for the benefit of patient treatment, care and safety.
- Provides optimal facilities focused in the learner’s development needs
- Maintains honesty and objectivity during assessment and appraisal.
- Shows willingness to participate in workplace-based assessments and demonstrates a clear understanding of their purpose.
- Recognises the importance of personal development as a role model to guide trainees in aspects of good professional behaviour.
- Shows willingness to advance own educational capability through continuous learning.

**C51. THE ORGANISATION OF NETWORKS AND THE BUILDING AND MAINTENANCE OF**

## RELATIONSHIPS, ENCOURAGING CONTRIBUTION TO AND WORKING WITH INTER-PROFESSIONAL TEAMS TO MEET THE BUSINESS OBJECTIVES.

**Applied Knowledge of:**

- The general principles of people management (see C49).
- Motivational techniques.
- Methods used to retain and develop staff to their full potential including the role of personal development plans (PDP).
- The principles and common practices of managing a team (line or matrix).

## Skills:

- To describe sources of appropriate feedback on performance and their relative importance, how to give feedback, and measure outcomes.
- To apply motivational techniques in reaching a project outcome.
- To apply leadership and motivational skills to management of multidisciplinary teams.
- To prepare notes, delegate, organise and lead teams.

## Behaviours:

- Recognises the importance of management / leadership style and influence on team dynamics and reaching departmental / project goals.
- Respects colleagues in a multidisciplinary environment.
- Recognises the needs to supervise the work of less experienced colleagues.

**C52. SUPPORTING THE SUCCESS OF THE ORGANISATION THROUGH ACTIVE CONTRIBUTION**

## TO DEVELOP STRATEGIC PLANS TO ACHIEVE GOALS, MANAGE RESOURCES AND PEOPLE, AND LEVERAGE PERFORMANCE.

**Applied Knowledge of:**

- The structure and processes of the pharmaceutical industry internal environment.
- The elements involved in the strategic assessment of a pharmaceutical product.
- The components required for the evaluation of an in-licensing / acquisition /collaboration option.
- The clinical, regulatory and commercial aspects for a product reclassification strategy.
- The identity of the industry’s key stakeholders in the external environment and how the industry’s activities impacts on them, including the general public.
- The roles of and relationship between the relevant national trade and professional organisations.

## Skills:

- To evaluate the commercial activities of a pharmaceutical company.
- To perform an analysis of industry key stakeholders, and the important relationships and interactions between them.
- To prepare for meetings – reading agendas, understanding minutes, action points and background research.
- To participate in succession planning and talent management.
- To distinguish between different ethical approaches in different situations and recognise the personal and professional challenges and responsibilities involved when making ethical judgements in the commercial environment.

## Behaviours:

- Recognises how internal business operations and drivers impact the relationships between the pharmaceutical industry and the wider healthcare environment.
- Recognises the degree of data requirements and the inherent limitations in the commercial analysis of pharmaceutical product potential.
- Recognises the commercial potential and limitations of in-licensing and collaborative options.
- Recognises the clinical (particularly safety-related) and commercial implications of pharmaceutical reclassifications.
- Recognises the relevance of a stakeholder analysis and its contribution to better relationships between them and improved communication of the industry’s activities.

**C53. ENSURING ORGANISATIONAL EXCELLENCE THROUGH DEVELOPMENT OF CRITICAL**

## EVALUATION SKILLS, ENCOURAGEMENT FOR IMPROVEMENT AND INNOVATION IN MANAGING CHANGE.

**Applied Knowledge of:**

- Role of team dynamics in the way a group, team or department functions.
- Facilitation and conflict resolution methods.
- Leadership styles and applicability to different situations and people.
- Business management principles; priority setting and production of a business plan.
- Commissioning, contracting and funding arrangements relevant to pharmaceuticals.
- Organisational performance management techniques and processes.
- The implications of change on systems and people.
- Project management methodology.
- Effective communication strategies and methods within organisations.
- Tools and techniques for managing stress.

## Skills:

- To use a reflective approach to practice with ability to learn from experience.
- To use assessment, appraisal and other feedback to understand and impart development needs.
- To recognise, analyse and know how to deal with unprofessional behaviours.
- To create open and non-discriminatory professional working relationships.
- To bring together different professionals and disciplines to provide high-quality multi-disciplinary teams.
- To communicate effectively in conflict resolution, providing feedback and identifying and rectifying team dysfunction.
- To facilitate, chair and contribute to meetings.
- To encourage staff to develop and exercise their own leadership skills.
- To identify and prioritise tasks and responsibilities including delegation and safe supervision.
- To contribute to staff development and training, including mentoring, supervision and appraisal.

## Behaviours:

- Recognises and respects diversity and differences in others.
- Recognises the importance of best practice, transparency and consistency.
- Recognises the need to manage time and to delegate.
- Is committed to participate in continuing professional development.
- Inspires confidence and trust and is committed to good communication.
- Takes shared responsibility for clinical performance, risk management and audit – in order to improve quality of the services.
- Is willing to articulate strategic ideas and use effective influencing skills.

**C54. IDENTIFICATION AND RECOGNITION OF STRENGTHS, DEFICIENCIES AND LIMITS TO ONE'S**

## KNOWLEDGE AND EXPERTISE.

**Applied Knowledge of:**

- Ways in which management styles impact on others.
- Methods of obtaining feedback from others.
- Tools and techniques for managing stress.
- Professional, legal and ethical codes of practice.
- The general principles of people management.

## Skills:

- To maintain and practise critical self-awareness to recognise stress in self and others, and to seek support as necessary.
- To balance personal and professional roles and tasks.
- To use a reflective approach to practice with ability to learn from experience.
- To use assessment, appraisal and other feedback to develop and understanding of own development needs.
- To take on differing and complementary roles within different areas of practice of pharmaceutical medicine.

## Behaviours:

- Recognises personal health as an important issue.
- Is prepared to accept responsibility.
- Is committed to participate in continuing professional development.
- Recognises the need to promote professional attitudes and values.
- Recognises the need for probity and the willingness to be truthful and admit errors.
- Recognises the needs for effective interaction with professionals in other disciplines and agencies.
- Respects the skills and contributions of colleagues.
- Recognises the limits of own professional competence and only practises within these.
- Recognises the importance of best practice, transparency and consistency.

**C55. CONTRIBUTION TO THE EFFECTIVE WORKING OF A HEALTHCARE TEAM OR OTHER**

## PROFESSIONAL GROUP.

**Applied Knowledge of:**

- Principles of management and leadership of people, business, industry R&D and medical function, product development and stakeholder groups (see C51-54 for detail):
  - People management;
  - Multidisciplinary team management, performance and change management;
  - Management and leadership in the pharmaceutical industry, project and product development management;
  - Business management, commissioning and contracting;
  - Stakeholder groups and trade and professional bodies - interaction and influencing;
  - Communication strategies, methods, activities, feedback and evaluation;
  - Personal management and leadership style and strengths – motivation, conflict, stress and self;

## Skills:

- To apply management, interpersonal, communication and leadership principles and practices to multi- disciplinary team-working in medicines development (se C51-54 for detail):
  - Reflective practice and critical self-awareness;
  - Feedback techniques, appraisal, assessment and review;
  - Motivational techniques;
  - Evaluation of commercial activities;
  - Stakeholder analysis;
  - Ethical judgement to commercial situations;
  - Run meetings;
  - Balance personal and professional roles;
  - Manage professionalism and professionals (recognise and apply equality and diversity);
- To facilitate training and development of others.
- To differentiate personal styles and preferences in interpersonal interactions and communications.
- To work with a wide range of people outside pharmaceutical medicine.
- To participate in succession planning and talent management.
- To undertake project planning and the meeting of allocated timelines for multidisciplinary roles.
- To relate time and time management to opportunity costs and other determinants of successful and resourceful project completion and outcome.

## Behaviours:

- Recognises and respects diversity and differences in others.
- Recognises the need to manage time and to delegate.
- Recognises the need for probity and the willingness to be truthful and admit errors.
- Recognises the needs for effective interaction with professionals in other disciplines and agencies.
- Recognises the need to supervise work of less experienced colleagues.
- Inspires confidence and trust, and is committed to good communication.
- Is willing to articulate strategic ideas and use effective influencing skills.
- Recognises the importance of management / leadership style and influence on team dynamics and reaching departmental / project goals.

**C56. EXPLANATION OF HIS/HER ACCOUNTABILITY TO STAKEHOLDERS, SOCIETY AND THE**

## DISCIPLINE OF PHARMACEUTICAL MEDICINE / MEDICINES DEVELOPMENT SCIENCES.

**Applied Knowledge of:**

- The structure, functions and socio-cultural value systems in which biomedical R&D takes place, and pharmaceutical medicine and medicines development science operates, as related to individual authority and accountability.

## Skills:

- Similar skills as described on C51-55 with focus on accountability as related to:
  - Communication;
  - Team-working and project and people management;
  - Inter-disciplinary work.

## Behaviours:

- Recognises need for responsibility and accountability for actions related to:
  - Patients and public;
  - Colleagues and management;
  - Ethics and professional bodies;
  - Legal and regulatory (auditors);
  - Sponsors, donors, payers;
  - Shareholders and market dynamics.
- Recognises the empowerment and accountability in the specific role as an opportunity for effective contributions to the organization and society at large.

**C57. APPLICATION OF QUALITY AND PERFORMANCE IMPROVEMENT CONCEPTS TO ADDRESS**

## ORGANISATIONAL PERFORMANCE ISSUES.

**Applied Knowledge of:**

- Principles of quality management and performance improvement.
- Time management and project management.
- Business management, priority-setting and production of a business plan.
- Organisational performance management techniques and processes.
- Risk management issues relevant to pharmaceutical medicine; sources of risk; risk management tools, techniques and protocols.
- The impact of government decisions on patient care, research and educational activities.
- The implications of change on systems and people.

## Skills:

- To relate time and time management to opportunity costs and other determinants of successful and resourceful project completion.
- To contribute to staff development and training, including mentoring, supervision and appraisal.
- To improve services / functions following evaluation / performance management.
- To work according to quality principles.

## Behaviours:

- - Recognises the importance of interpersonal skills for Pharmaceutical Physicians and MDS to influence / drive project / team outcomes.
  - Recognises the importance of effective time management and project management to meet project and personal goals.
  - Adopts a patient-focused approach to decisions and acknowledges the rights, values and strengths of patients and the public.
  - Is committed to participate in continuing professional development.
  - Recognises the need for transparency and the willingness to be truthful and admit errors.

**STATEMENT OF COMPETENCE FOR PHARMACEUTICAL PHYSICIANS AND DRUG DEVELOPMENT SCIENTISTS**

## The Pharmaceutical Physician/Drug Development Scientist:

- **Is able to identify unmet therapeutic needs, evaluate the evidence for a new candidate for clinical development and design a Clinical Development Plan for a Target Product Profile.**
- **Is able to design, execute and evaluate exploratory and confirmatory clinical trials and prepare manuscripts or reports for publication and regulatory submissions.**
- **Is able to interpret effectively the regulatory requirements for the clinical development of a new drug through the product life-cycle to ensure its appropriate therapeutic use and proper risk management.**
- **Is able to evaluate the choice, application and analysis of post-authorization surveillance methods to meet the requirements of national/international agencies for proper information and risk minimization to patients and clinical trial participants.**
- **Is able to combine the principles of clinical research and business ethics for the conduct of clinical trials and commercial operations within the organization.**
- **Is able to appraise the pharmaceutical business activities in the healthcare environment to ensure that they remain appropriate, ethical and legal to keep the welfare of patients and research participants at the forefront of decision making in the promotion of medicines and design of clinical trials.**
- **Is able to interpret the principles and practices of people management and leadership, using effective communication techniques and interpersonal skills to influence key stakeholders and achieve the scientific and business objectives.**

69


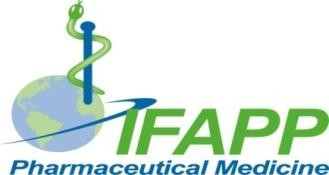

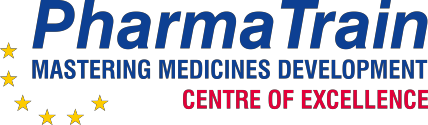

Supplement: Supplementary file 1 [file Data_Sheet_1.docx]
